# The diversification and lineage-specific expansion of nitric oxide signaling in Placozoa: insights in the evolution of gaseous transmission

**DOI:** 10.1038/s41598-020-69851-w

**Published:** 2020-08-03

**Authors:** Leonid L. Moroz, Daria Y. Romanova, Mikhail A. Nikitin, Dosung Sohn, Andrea B. Kohn, Emilie Neveu, Frederique Varoqueaux, Dirk Fasshauer

**Affiliations:** 1 Whitney Laboratory for Marine Bioscience and Departments of Neuroscience, University of Florida, St. Augustine and Gainesville, FL 32080 USA; 2 Institute of Higher Nervous Activity and Neurophysiology, Russian Academy of Sciences, Moscow, 117485 Russia; 3 Belozersky Institute of Physico-Chemical Biology, Moscow State University, Moscow, 119991 Russia; 4 Department of Fundamental Neurosciences, University of Lausanne, 1005 Lausanne, Switzerland

**Keywords:** Metabolomics, Oxidoreductases, Metabolomics, Small molecules, Evolutionary biology, Genetic markers, Biochemistry, Evolution, Neuroscience, Zoology

## Abstract

Nitric oxide (NO) is a ubiquitous gaseous messenger, but we know little about its early evolution. Here, we analyzed NO synthases (NOS) in four different species of placozoans—one of the early-branching animal lineages. In contrast to other invertebrates studied, *Trichoplax* and *Hoilungia* have three distinct NOS genes, including PDZ domain-containing NOS. Using ultra-sensitive capillary electrophoresis assays, we quantified nitrites (products of NO oxidation) and l-citrulline (co-product of NO synthesis from l-arginine), which were affected by NOS inhibitors confirming the presence of functional enzymes in *Trichoplax*. Using fluorescent single-molecule in situ hybridization, we showed that distinct NOSs are expressed in different subpopulations of cells, with a noticeable distribution close to the edge regions of *Trichoplax*. These data suggest both the compartmentalized release of NO and a greater diversity of cell types in placozoans than anticipated. NO receptor machinery includes both canonical and novel NIT-domain containing soluble guanylate cyclases as putative NO/nitrite/nitrate sensors. Thus, although *Trichoplax* and *Hoilungia* exemplify the morphologically simplest free-living animals, the complexity of NO-cGMP-mediated signaling in Placozoa is greater to those in vertebrates. This situation illuminates multiple lineage-specific diversifications of NOSs and NO/nitrite/nitrate sensors from the common ancestor of Metazoa and the preservation of conservative NOS architecture from prokaryotic ancestors.

## Introduction

Nitric oxide (NO) is a versatile gaseous transmitter widely distributed among prokaryotes and eukaryotes^1–4^. Multiple functions of this messenger are direct reflections of the free-radical nature of NO and, subsequently, its complex free radical chemistry^5^. Dissolved NO passes readily across membranes and diffuses into neighboring cells interacting with many biological molecules, including DNA, lipids, proteins^5^ with several specialized receptors such as guanylate cyclases^6–8^. Thus, NO can act as a volume transmitter locally (i.e., its release is not restricted by the synaptic cleft as for many classical neurotransmitters), and it is easily converted into nitrite and nitrate by oxygen and water. In cells, the synthesis of NO is catalyzed by the enzyme NO synthase (NOS) through a series of complex redox reactions by the deamination of the amino acid l-arginine to l-citrulline. The reaction requires the presence of oxygen, as a precursor, and NADPH^5^. The large enzyme operates as a dimer and consists of two enzymatic portions, an oxygenase domain that binds heme and the redox factor tetrahydrobiopterin (H4B) and a reductase domain that is related to NADPH-dependent microsomal cytochrome P450^9^.

The role and mechanism of NO signaling are well studied in mammals. However, little is known about the early evolution of NO signaling in animals, mostly due to limited comparative data from basally branching metazoans, including Cnidaria, Porifera, Ctenophora, and Placozoa.

Among other things, NO is involved in feeding, chemosensory processing, and locomotion of such cnidarians as *Hydra* and *Aglantha*^10–13^, where NO-dependent communications were likely mediated by just one type of NO synthase (NOS)^1^. In the sponge *Amphimedon*, only one NOS gene has been identified^14^. NO-cGMP signaling has been implemented in the regulation of larval settlement^15^ and rhythmic body contractions^16^. In the ctenophore, *Mnemiopsis leidyi,* again, only one NOS gene has been recognized so far^17^, but the functional role of NO has not been studied. Interestingly, in another ctenophore species, *Pleurobrachia bachei*, NOS appears to have been lost^18^.

Nothing is known about the presence and the distribution of NO signaling in Placozoa—an important but little-studied lineage of cryptic marine animals. The current consensus stands that Placozoa is the sister group to the clade Cnidaria + Bilarteria^18–20^, although some authors consider Placozoa as highly derived and secondarily simplified cnidarians^21^. Regardless of the proposed phylogenies, Placozoa represents a crucial taxon to understand the origin and evolution of animal traits and the nervous system in particular^22^.

Placozoans, such as *Trichoplax*, *Hoilungia,* and other cryptic species (most of them are not formally described and known as haplotypes)^23,24^, are the simplest known free-living animals with only six morphologically recognized cell types organized in three layers^25^. Nevertheless, *Trichoplax* has quite complex behaviors^26–29^, including social-like patterns^30^. Here, we biochemically showed that *Trichoplax* exhibits functional NOS activity, and, in contrast to other pre-bilaterian animals, placozoa independently evolved three distinct NOSs (as vertebrates) with a profound diversification of NO-cGMP signaling components, and likely the capabilities of nitrite/nitrate sensing by distinct NIT domain-containing guanylate cyclases, which represents a remarkable example of the evolution of gaseous transmission in the animal kingdom.

## Results

### Comparative analysis of NOSs

**Figure 1:**
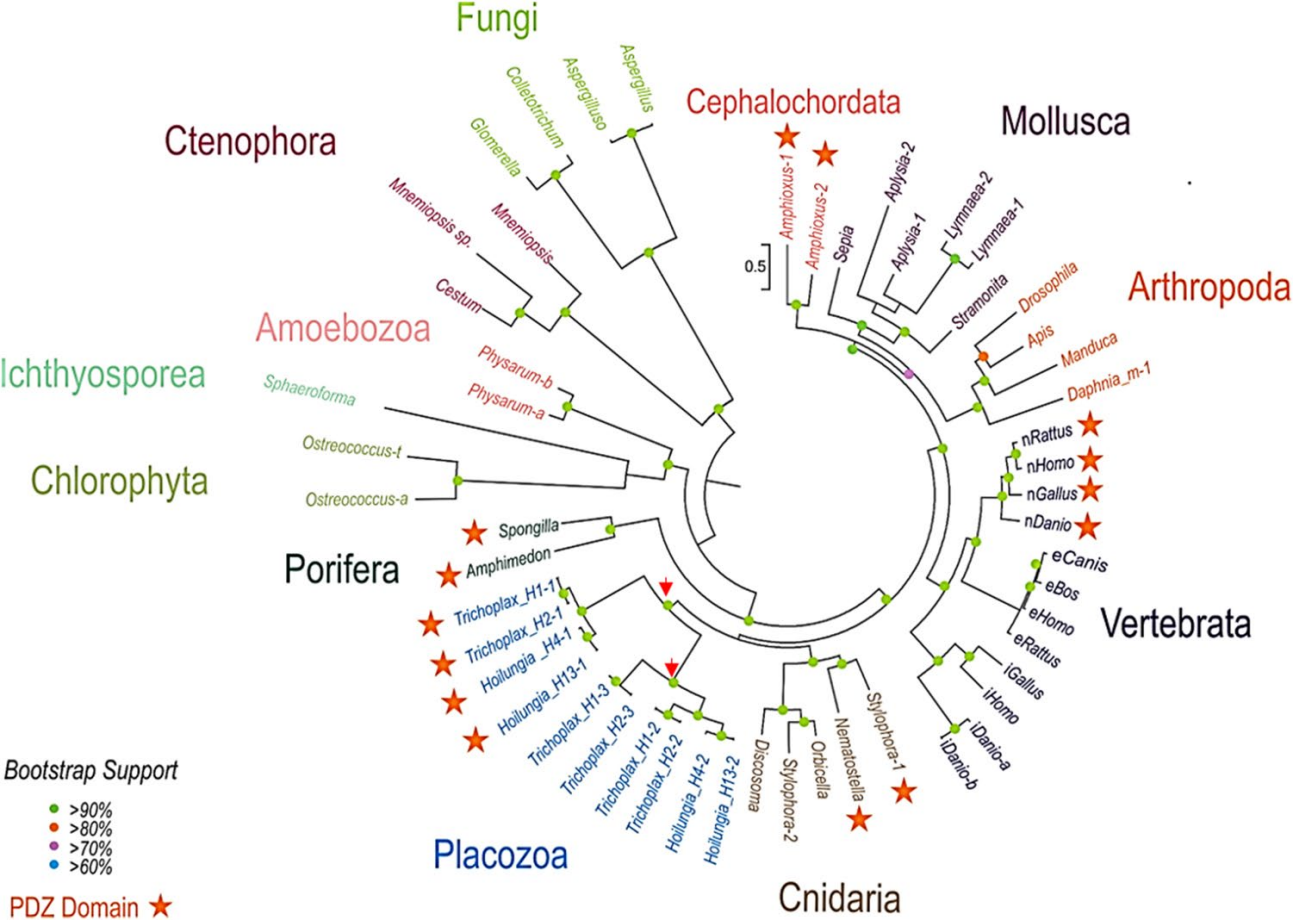
The diversity and evolutionary relationships of nitric oxide synthases in animals and other eukaryotes. The representative lineages of deuterostomes, protostomes, and basal metazoans are highlighted. Unicellular eukaryotes and green algae are shown as outgroups. Names of the species are indicated in each case using the existing classification of NOSs (see text for details). For vertebrates, we marked different NOS to reflect their functional specification. nNOS—neuronal, which corresponds to human PDZ-containing NOS-1; iNOS—inducible or NOS-2; eNOS—endothelial or NOS-3. For placozoans, we indicated the haplotype (H1, H2, H4, H13)/species name and the number of NOS genes as referred to in the text (e.g., they correspond to *Trichoplax* NOS-1 [PDZ-domain containing], NOS-2 and NOS-3 respectively). All NOSs containing PDZ domains are indicated by asterisks. Phylogenetic trees were inferred using the Maximum Likelihood algorithm. The references for each particular gene and/or their sequences with relevant GeneBank accession numbers are summarized in the supplementary dataset (Excel Table).

**Figure 2:**
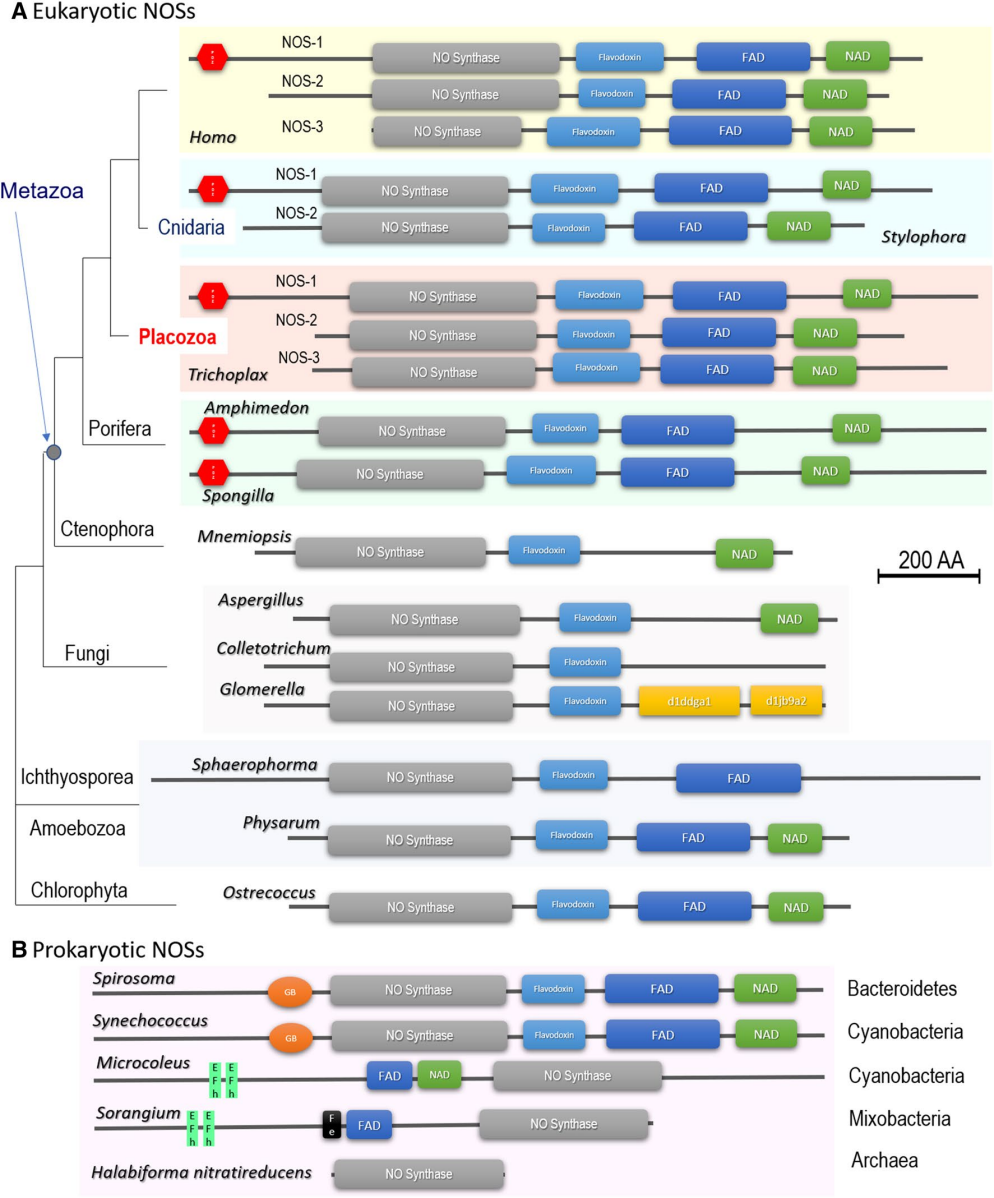
Conservative domain organization of eukaryotic (**A**) and prokaryotic (**B**) NOS. All NOS are presented on the same scale, including the sizes of all domains and proteins. Grey box—NOS oxygenase domain (Pfam NO_Synthase PF02898); light blue box—Flavodoxin_1 (Pfam PF00258); dark blue box—FAD_binding_1 domain (Pfam PF00667); green box—NAD_binding_1 (Pfam PF00175). (**A**) Eukaryotic NOSs include representatives of 5 metazoan phyla and 4 non-metazoan lineages with their respective phylogenetic relationships and species names (see also Fig. 1 for details). Three human (*Homo*) NOSs with PDZ domain (red hexagon) containing neuronal NOS-1 are at the top of the figure. *Trichoplax* is the only know invertebrates with three NOS genes, also including PDZ domain-containing NOS-1. The ctenophore *Mnemiopsis* and all thee fungal NOSs apparently lost the FAD domain. *Colletotrichum*, *Glomerella,* and *Sphaerophorma* lost NAD domain. Interestingly, NOS in *Glomerella* contained unusual domains absent in other NOSs. (**B**) Two prokaryotic NOSs (*Spirosoma* and *Synechoccus*) have the canonical 4 domain organization, but with specific heme-binding globin domain (Pfam PF00042). The cyanobacteria *Microcoleus* and myxobacteria *Sorangium* apparently have a reverse order of canonical domains and two distinct calcium-binding motifs (EF-hand, SMART SM000054). *Sorangium* NOS also has BFD-like [2Fe-2S] iron-binding domain (Pfam PF04324). The Archaea representative (*Halabiforma*) has an NOS oxygenase domain only. The references for each particular gene and/or their sequences with relevant GeneBank accession numbers are summarized in the supplementary dataset (Excel Table).

Figure 1 shows the phylogenetic relationships among different animal NOSs, where representatives of all basal metazoan lineages form distinct branches for their respected NOSs with evidence for relatively recent duplication events consistent to an early origin and diversification of NOSs in other eukaryotic groups including Amebozoa and Fungi as sister lineages to Metazoa. Figure 2 illustrates the domain organization of NOSs, which is conservative in animals and sufficiently diverse in other eukaryotic groups. We did not find the evidence for NOS in choanoflagelates sequenced so far, including *Monosiga* with the sequenced genome. Choanozoa, the phylogenetically closest taxon to Metazoa^31^, might have lost NOS from its eukaryotic ancestor. NOS was also lost in the nematode *C. elegans*.

Interestingly, ctenophores, known as the sister lineage to the rest of metazoans^19,20,32^, have only one highly derived NOS without flavodoxin domain (Fig. 2) as represented by two *Mnemiopsis* species and *Cestum* in the tree (Fig. 1); and it remains to be determined whether ctenophores possess functional NO-producing enzyme(s).

All metazoan NOSs have a relatively conservative domain architecture, whereas many eukaryotic lineages lost one or more domains (Fig. 2); it might be possible due to the parasitic lifestyle of some fungi and *Sphaerophorma*. Representatives of such early-branching lineages as Amoebozoa and green algae do possess four major canonical NOS domains. Surprisingly, we also found that in some prokaryotic NOSs have the very same domain organization as in the animal and algal NOS genes (Fig. 2). This finding strongly suggests that the complex multidomain NOS architecture was present in the common ancestor of all eukaryotes. All studied invertebrates have only one or two NOS genes, which do not directly correspond to the well-established vertebrate subfamilies of the enzymes^1^. In contrast, we identified three distinct NOS genes in the *Trichoplax* genome (haplotype H1—Fig. 2 and Supplement 1), and three other placozoan species or haplotypes H2, H4, H13, and one of the NOSs contains the PDZ domain similar to the mammalian neuronal NOS.

The presence of PDZ domain-containing NOSs is a distinct feature of all four placozoan species sequenced so far (NOS1 in H1, H2, H4, and H13). H1 and H2 represent the classical *Trichoplax* genus^23^, while H4 and H13 belong to the newly described genus *Hoilungia*^24,33^. The clustering of NOSs in placozoans reflects their phylogenetic relationships stressing that H4 and H13 (*Hoilungia*) vs. H1 and H2 (*Trichoplax*) belong to different lineages.

The PDZ domain and N-terminal motifs are required for the anchoring of NOS to plasmatic or intracellular membranes, subcellular localization, and integration to many signaling components like in the mammalian neuronal nNOS^9,34–37^. nNOS is different from the two other mammalian isoforms as its N-terminal PDZ domain can heterodimerize with the PDZ domains of postsynaptic density proteins (e.g., PSD95) or syntrophin^38^ and others^9^. Thus, we might suggest similar molecular functions in *Trichoplax* and *Hoilungia*.

The rate of evolution of the PDZ domain-containing NOSs is comparable to other NOSs for all placozoan species. The branching patterns of NOS trees (Fig. 1) reveals that three NOSs in Placozoa are the results of two independent duplication events from the common placozoan ancestor (red arrows in Fig. 1). The first splitting separated NOS1, and the second, more recent split led to NOS2 and NOS3.

Of note, we also identified two NOSs in the stony coral *Stylophora*, which has one NOSs with the PDZ domain (Figs. 1, 2), and a PDZ domain was detected upstream of the *Nematostella* NOS gene in the existing genome assembly. Also, two sponges (*Amphimedon* and *Spongilla*) possess PDZ-containing NOS. As the PDZ domains of NOSs appear to be homologous, it should be investigated whether the PDZ domain-containing form represents the ancestral form in animals. In contrast, ctenophores and many other animal lineages (Fig. 1) do not have PDZ-containing NOS genes, which might also be truncated (Fig. 2).

**Figure 3:**
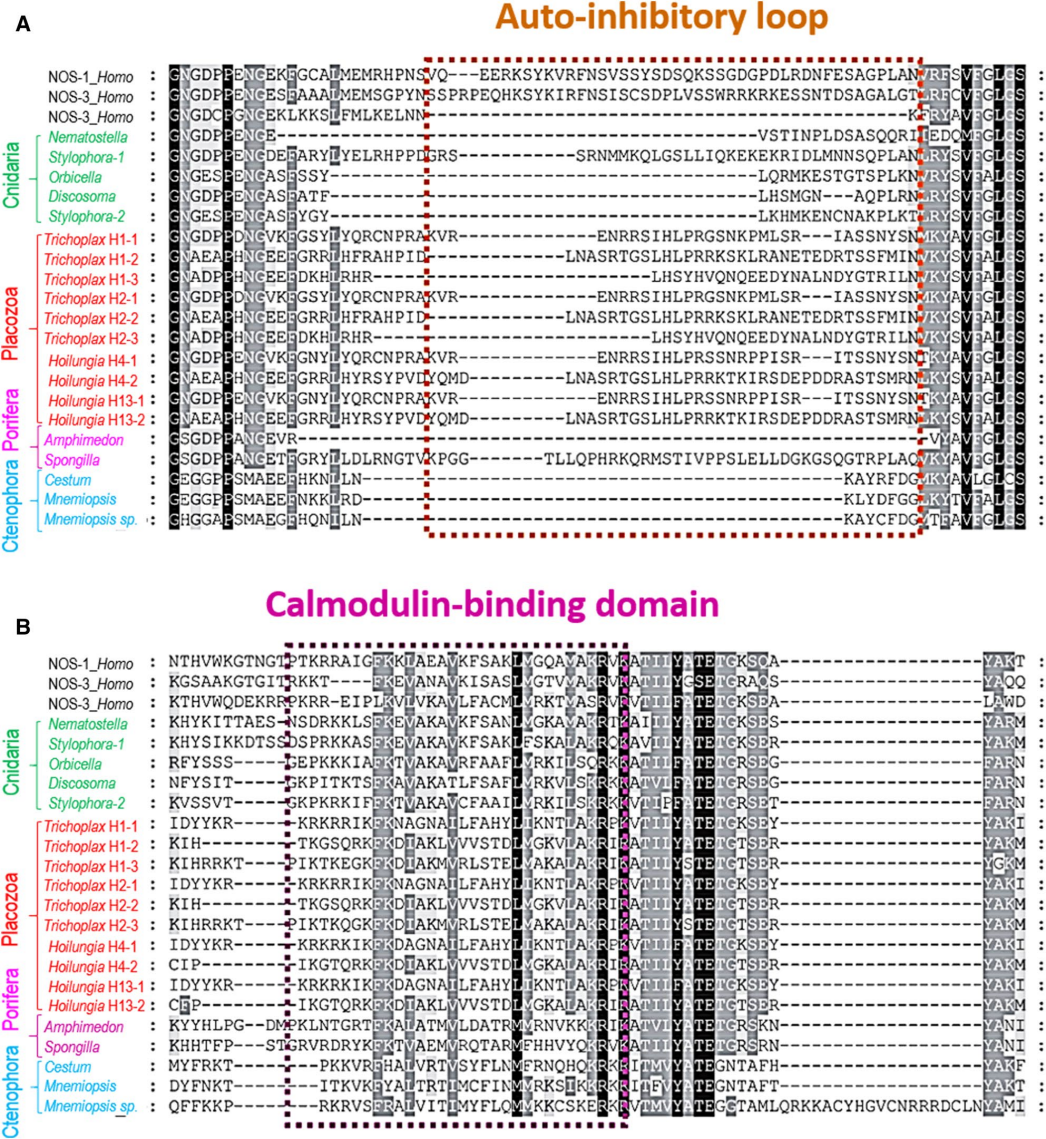
Auto-inhibitory N-terminal inserts in NOSs (**A**) and calmodulin-binding domain (**B**): Insights into controlling Ca^2+^-dependence and the conservation of calmodulin-binding sites in placozoans. Alignments of selected NOSs was performed using the sequence information from species outlined in Fig. 1. The references for each particular gene and/or their sequences with relevant GeneBank accession numbers are summarized in the supplementary dataset (Excel Table).

NOS is a complex enzyme (Fig. 2 and Supplement 1) requiring several co-factors for its activation, and Ca^2+^-dependence of different NOSs in mammals is determined by the presence of the autoinhibitory inserts and calmodulin-binding sites^39–46^. Figure 3 shows the presence/absence of such motifs and the auto-inhibitory loops across basal metazoan lineages. The canonical human Ca^2+^-independent iNOS lacks such a loop; it is bound to calmodulin (CaM) in a Ca^2+^-independent manner. Of note, calmodulin is one of the most abundantly expressed genes in *Trichoplax*.

Mammalian iNOS activation is often induced by lipopolysaccharides as a part of innate immunity responses on bacterial infection^4^. Ctenophores, the demosponge *Amphimedon*, *Nematostella,* and three coral NOSs also lack the auto-inhibitory loop (Fig. 3) and could be Ca^2+^-independent and, apparently, inducible (e.g., by bacteria or during development and differentiation). However, all three *Trichoplax* and *Hoilungia* NOSs contains an intermediate size insert in this position (Fig. 3 and supplement 1): these NOSs might be dormant or, partially inducible. Thus, the direct detection of endogenous enzymatic activity is needed to validate NOS expression, which we performed using direct microchemical assays.

### Detection of endogenous NOS activity in *Trichoplax*

Because some NOSs can be inducible or pseudogenes, the molecular/sequence information itself is not sufficient for the demonstration of NOS activity. Thus, we implement two complementary approaches to confirm the presence of functional NOSs in placozoans.

#### Arginine/citrulline assays

NO is known to be produced enzymatically from molecular oxygen and l-arginine with l-citrulline as the co-product^5^. It was interesting that all NOS-related metabolites were detected in *Trichoplax* at relatively high concentrations, 0.35 mM for arginine, and 0.5 mM for citrulline.

However, absolute concentrations of arginine and citrulline per se are not a direct indicator of NOS activity. On the other hand, the Arginine/Citrulline ratio and the sensitivity of this ratio to NOS inhibitors is a reliable assay for the presence of functional NOS, which is also validated in different species^47,48^.

**Figure 4:**
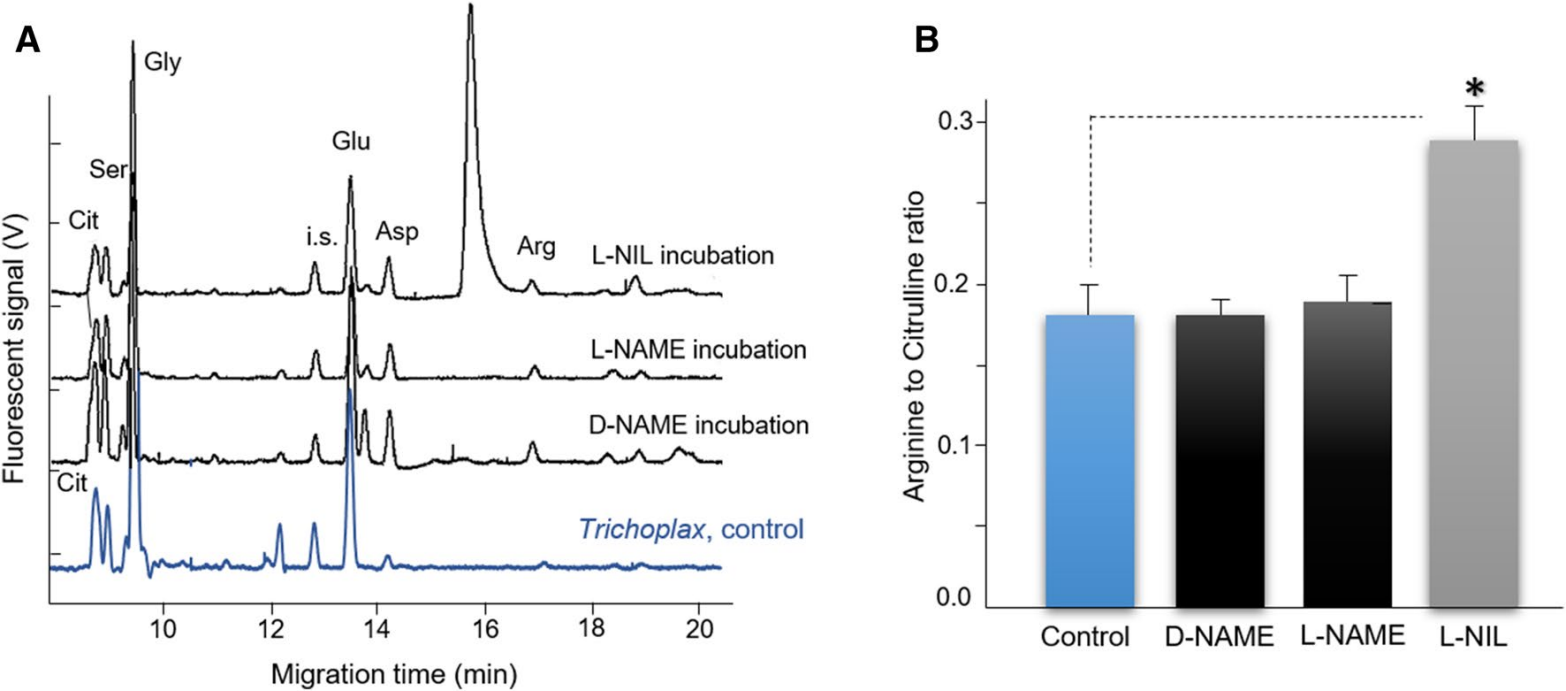
Detection of NOS amino acid-derived metabolites by capillary electrophoresis and their sensitivity to NOS inhibitors. (**A**) Electropherograms of individual animal samples and l-Arginine to l-Citrulline ratios of *Trichoplax adhaerens* following treatment with NOS inhibitors. Arginine and citrulline peaks were identified with spike standards and shown as Arg and Cit, respectively. i.s.—internal standards (see “Materials and methods” for details). Samples were loaded using electrokinetic injection (8 kV for 12 s) and then analyzed under a stable 20 kV voltage at 20 °C in 50 μm I.D. and 360 μm O.D. capillary with 30 mM borate/30 mM SDS, pH 10.0. Electropherograms of *Trichoplax* incubated with *N*^G^-nitro-l-arginine methyl ester or L-NAME (500 μM), D-NAME (500 μM), and L-N^6^-(1-iminoethyl)-lysine, L-NIL (1 mM), for 30 min at room temperature. (**B**) Arginine-to-Citrulline ratio of *Trichoplax* after treatment with putative NOS inhibitors; only L-NIL induced statistically significant increase of Arg/Cit ration suggesting the reduction of l-citrulline production (n = 5, data presented as mean ± SD), Student’s t test, *p < 0.05, treated (NOS inhibitor/L-NIL) vs. control group using paired t-test; see results for details.

Using a highly sensitive capillary electrophoresis (CE) microchemical assay with attomole detection limits, we demonstrated that *Trichoplax* produced l-citrulline, and its production is also reduced by NOS inhibitors (Fig. 4). It was expected from experiments on vertebrates and mollusks^48–50^ that the arginine-to-citrulline ratio would increase after *Trichoplax* was incubated in either L-NAME or L-NIL. The arginine-to-citrulline ratio increased by twofold in the case of L-NIL (Fig. 4). However, there was only a small increase with L-NAME, indicating L-NIL effectively inhibited the NOS enzyme as in mollusks^48–50^, but L-NAME did not. The reason for this difference might reflect differences in either l-arginine uptake, which might be blocked by arginine analogs or distinct enzymatic regulation of NOS in placozoans, or nonenzymatic interference of these inhibitors with NO production^51^. Combined, these CE/microchemical data indicate that placozoans have a substantial level of endogenous NOS activity.

#### Nitrite assays

Due to rapid NO oxidation in biological tissues^5^, NO_2_^−^ is considered as the most reliable reporter of functional NOS. In contrast, more stable (and less dynamic) terminal oxidation products of NO—nitrates (NO_3_^−^) cannot be used for these purposes since they can also be accumulated from various food sources. Thus, by employing CE with the conductivity detection, we provided the additional direct evidence for endogenous NOS activity using nitrite (NO_2_^−^) assay^50,52^.

NO oxidation metabolites were monitored, and concentrations were derived from in vitro calibration curves prepared from standard solutions of nitrate and nitrite at various concentrations (10 nM–500 μM). With the regression equations, the limit of detection (LOD) of nitrate was determined to be 13.3 nM for nitrite and 32.4 nM for nitrate. These LODs were sufficient to quantify nitrite and nitrate in *Trichoplax*.

**Figure 5:**
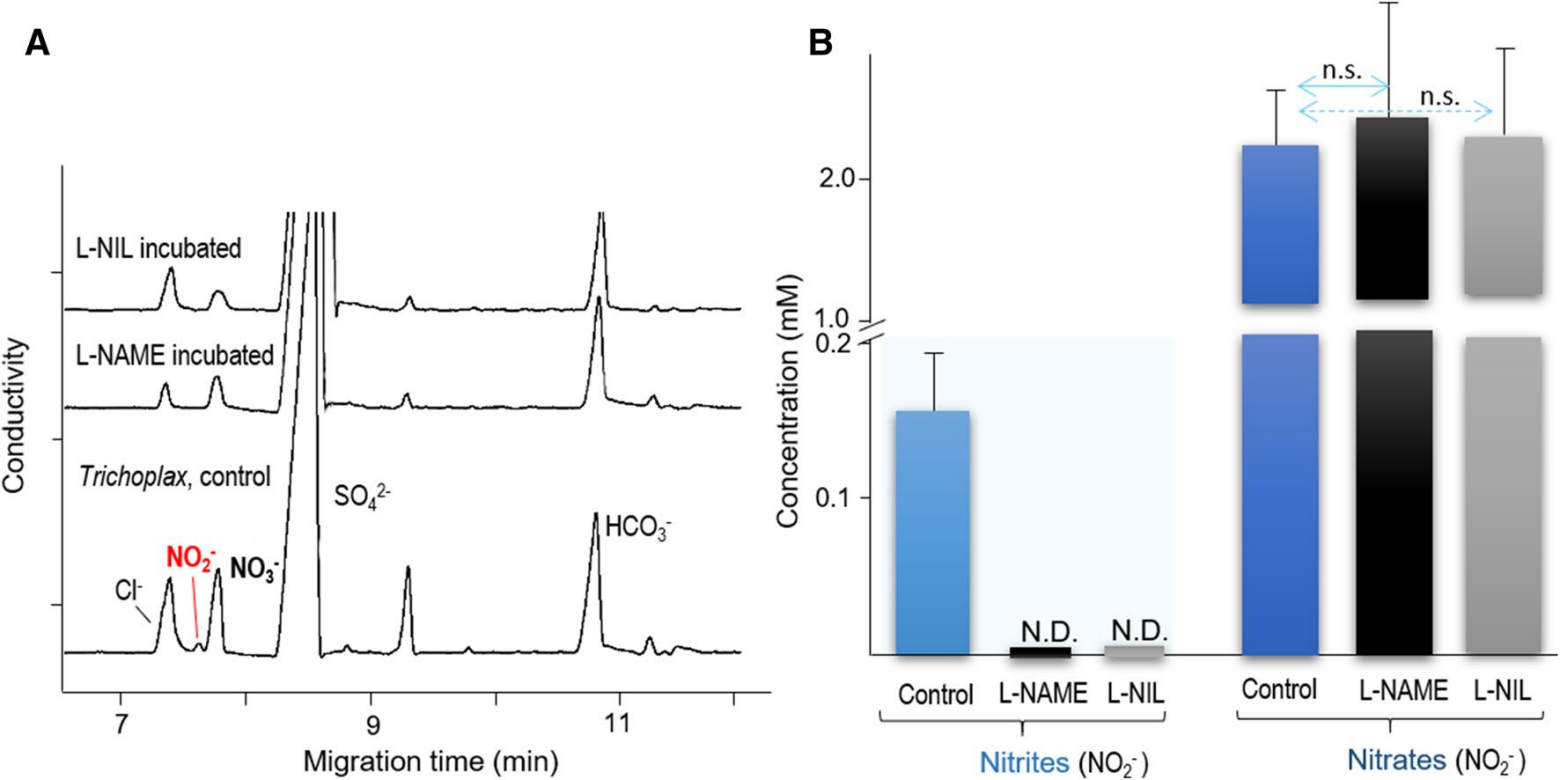
Detection of products of NO oxidation (NO_2_^−^ and NO_3_^−^) by capillary electrophoresis and their sensitivity to NOS inhibitors. Nitrites, products of NO oxidation, were detected in all control samples and were undetected (N.D.) following NOS inhibitor incubation (see text for details). The separation was conducted in a 75 cm length of 50 μm I.D. and 360 μm O.D. capillary with arginine/borate buffer, pH 9.0. All samples were loaded using electrokinetic injection (− 1 kV for 12 s), and then analyzed under a stable − 15 kV voltage at 20 °C. (**A**) Electropherograms of *Trichoplax* only, and *Trichoplax* incubated for 30 min with *N*^G^-nitro- l-arginine methyl ester or L-NAME (500 µM), and L-N^6^-(1-iminoethyl)-lysine or L-NIL (1 mM). (**B**) Nitrite and nitrate concentration profiling after 30 min of NOS inhibition (n = 5). Data are presented as mean ± SD; bars show control vs. treated (NOS inhibitors) group; ns: not significant (Student’s t test).

Surprisingly, we found that *Trichoplax* contains high micromolar concentrations of NO_2_^−^, which were undetectable, within 30 min, after the treatment by NOS inhibitors such as L-NAME and L-NIL (Fig. 5). In control *Trichoplax*, about 150 µM nitrite was detected, but after the incubation of animals with the NOS inhibitors, no nitrite was observed, suggesting the suppression of endogenous NOS activity (Fig. 5).

### The expression and distribution of NOS in *Trichoplax*

Fixative-resistant NADPH-diaphorase (NADPH-d) histochemistry has been reported as a marker of functional NOS in both vertebrates and invertebrates^50,53–56^. Here, we employed this assay for the initial screening of the NOS expression in *Trichoplax adhaerens* (H1) and its related species *Hoilungia hongkongensis* (H13)^33^. The NADPH-d histochemical activities in both placozoans were noticeable weaker (an order magnitude less) compared to the majority of other vertebrate and invertebrate species studied using the same protocol^13,57–62^. We noted that the intensity of NADPH-d labeling was similar to those described in the pelagic pteropod mollusk *Clione limacina*, where NO controlled swimming^63^. No contamination from algae was detected using the careful microscopic examination.

**Figure 6:**
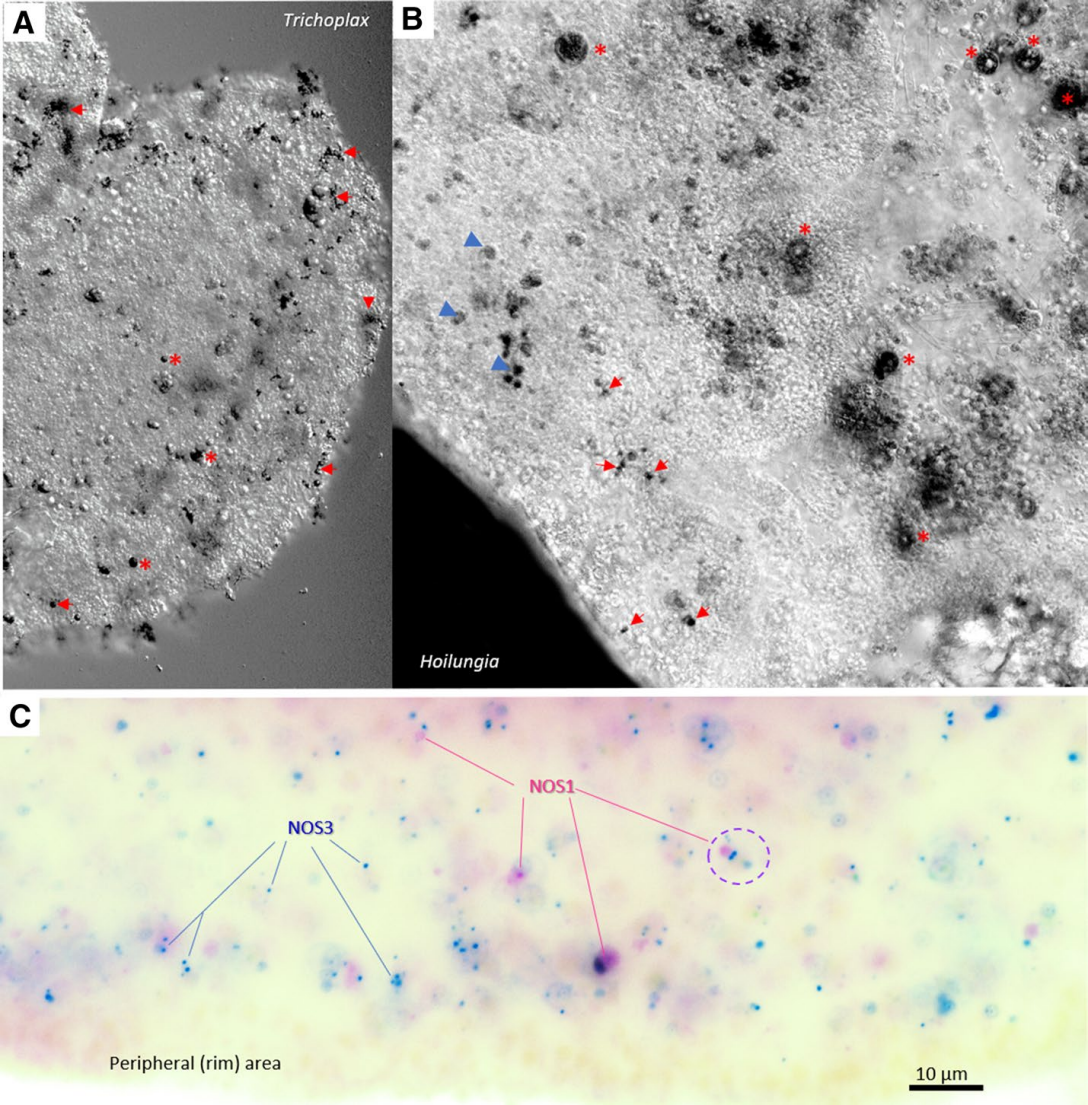
NOS expression in Placozoans. (**A**,**B**) NADPH-diaphorase histochemistry and the distribution of putative nitrergic cells in two species of Placozoa: *Trichoplax adhaerens* (**A**) and *Hoilungia hongkongensis* (**B**). NADPH-d reactive cells (black) are broadly distributed across the animal. In both species, relatively large cells (asterisks) correspond to so-called “shiny spheres,” whereas the arrows indicate an example of different NADPH-d reactive cells with some tendencies of their distribution close to the edge of animals. (**C**) Expression of two NOSs in *Trichoplax* using single molecules fluorescent in situ hybridization (smFISH, pseudo-color). Blue dots—NOS-3 and purple dots—PDZ-containing NOS-1. A dotted circle indicates an example of a cell where both NOS are co-localized within the same cell. Note, NOS-expressing cells do not occur at the very edge of the animal. Scale: 10 µm.

We revealed very similar NADPH-d labeling patterns in both *Trichoplax* and *Hoilungia* (Fig. 6A,B). There were several large (> 10 µm) structures; some of them correspond to the so-called “shiny spheres”^64^ and numerous small (4–6 µm) NADPH-d reactive cells were broadly distributed over different parts of the animal including the upper epithelial layer and, in some cases, close to crystal cells, known to be gravity sensors^65^. We estimate that about 2% of placozoan cells might be NADPH-d reactive. These cells might be candidates for NOS-containing (NO-releasing) cells. However, NADPH-d histochemistry cannot distinguish different NOS isoforms.

### Single-molecule in fluorescent in situ hybridization (smFISH)

Next, we used sequences for both NOS1 and NOS3 to characterize their expression and distribution in *Trichoplax adhaerens* by single-molecule FISH (smFISH) as the most sensitive assay for this purpose (we did not detect an expression of NOS2 in *Trichoplax* transcriptomes^66^). In both cases, we observed the cell-specific distribution of distinct NOS isoforms (Fig. 6C). Most of the NOS-containing cells were broadly distributed (similar to NADPH-d reactivity, but ‘shiny spheres” were not labeled by in situ hybridization probes). It appears that PDZ containing NOS1 had higher levels of expression than NOS3, and only partial co-localization of the two NOSs in the same cells was observed (Fig. 6C). We also noted that the NOSs are not located to the most peripheral cell layer but found in cells close to the rim. Due to a relatively high level of endogenous fluorescence in the central part of the animal, the precise cell identity of NOS-positive cells was difficult to determine. However, we noticed that both NOS could be co-localized in a very small subset of cells close to the edge of these disk-like animals.

### NO targets: diversification of cGMP signaling and identification of NIT (nitrite/nitrate sensing) domains in Placozoa and other metazoans

NO can act via cyclic guanosine monophosphate or cGMP as a second messenger. In this signaling pathway, NO binds to the heme group of soluble guanylate cyclases (sGCs), member of the adenylyl cyclase superfamily^67,68^, with a characteristic catalytic CYC domain, leading to the increase of cGMP synthesis^6–8,69–73^; by binding to ATP, sGC can also couple NO signaling to cellular metabolism^74^. Three groups of potential NO sensors have been identified in placozoans.

**Figure 7:**
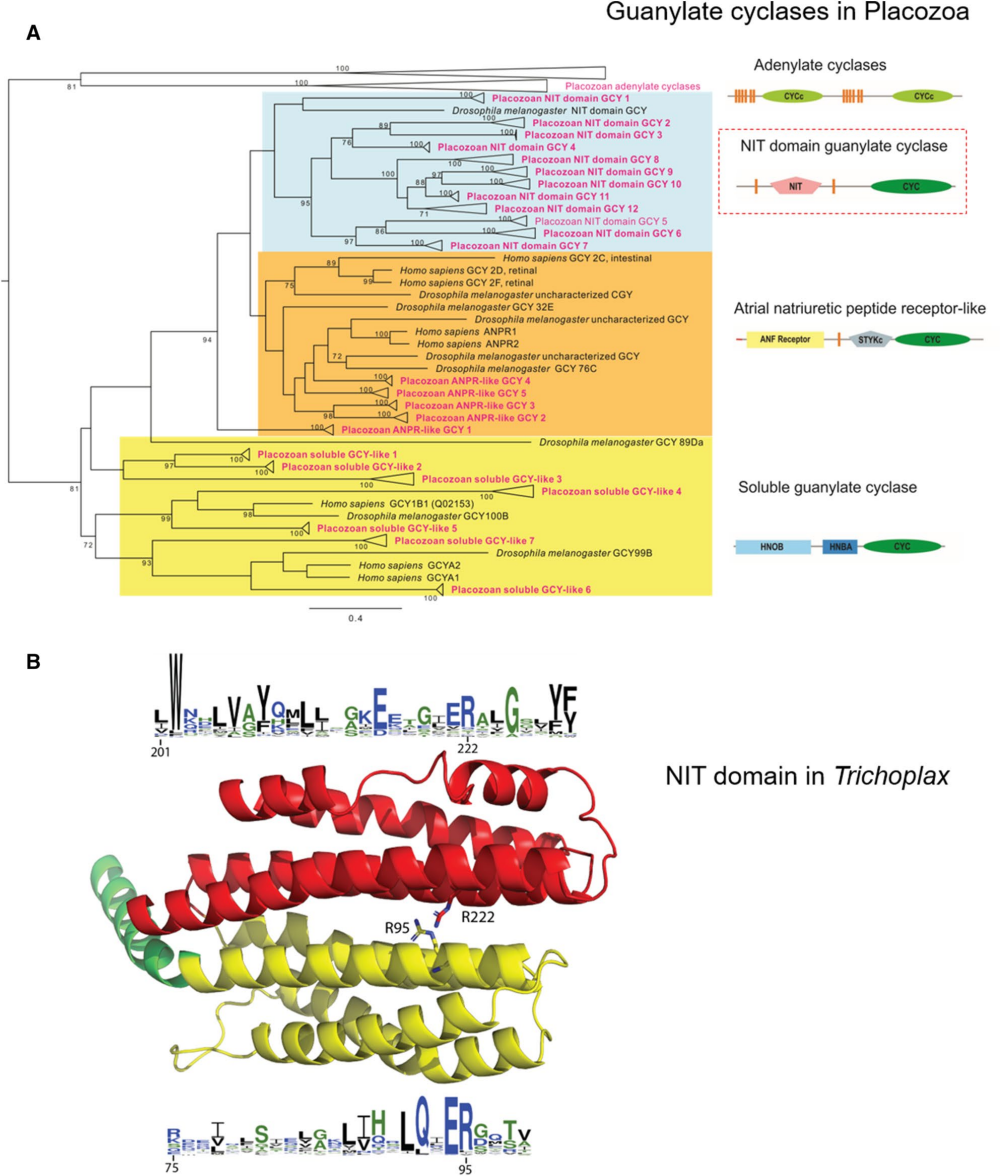
The diversity and lineage-specific expansion of sGC and related NO receptors in placozoans. (**A**) Maximum likelihood phylogenetic tree of placozoan soluble guanylate cyclases (sGC) and two groups of related enzymes: Atrial Natriuretic Peptide-like receptors (ANPRs), some of which contain unusual NIT domains—putative nitrite/nitrate sensing receptors (see text), and adenylate cyclases as outgroups. 119 protein sequences (the supplementary dataset (excel Table)) were trimmed down to cyclase domains and produced an alignment 325 aa long. Alignment was analyzed in IQTREE^105^ using LG + I + G4 evolution model chosen automatically with Bayesian information criterion. Tree robustness was tested with 2,000 replicates of ultrafast bootstrap. Orthologous proteins from 4 placozoan species (red text) were analyzed, and their branches collapsed in the tree: *Trichoplax adhaerens* (H1), *Trichoplax* sp. (H2), *Hoilungia* sp. (H4) and *Hoilungia hongkongensis* (H13), except for adenylate cyclases that were only from *Trichoplax adhaerens,* and NIT domain GCY3 which were found only in *Hoilungia* genus. Human and *Drosophila* orthologs are shown. The domain organization of three groups of predicted guanylate cyclases in placozoans is also schematically illustrated. The full (uncollapsed) version of this tree can be found in Supplemental Figure S1. (**B**). NIT domains in placozoans. The putative nitrate- and nitrite-sensing NIT domains of animals are homologous to prokaryotic NIT domains. Phyre2 was used to generate a structural model for the NIT domain of 007393 from *Trichoplax adhaerens*. The Phyre model is mostly based on the structure NIT domain of the NasR transcription antiterminator (pdb ID: 4AKK). The NIT domain consists of two four-helix bundles, shown in yellow and red. At their interface, two conserved arginines are thought to be involved in ligand binding. The sequence conservation of the two helices at the interface is shown by a webLogo representation^106^. The overall height of a stack indicates the sequence conservation at a certain position, whereas the height of symbols within the stack indicates the relative frequency of each amino acid at that position.

First, the *Trichoplax* and *Hoilungia* genomes encode seven sGCs (Fig. 7A), whereas only three orthologs were identified in humans. All these enzymes have the canonical heme NO binding domain and associated domain organization, and the predicted sGCs from placozoans form clusters appropriately with the α and β sGCs of humans. The heme-dependent NO sensor HNOBA (PF07701) was also found associated with sGC^71,75^.

Second, we identified in *Trichoplax* and their kin membrane-bound NO receptor candidates (Fig. 7A). *Trichoplax* also has five orthologs of atrial natriuretic peptide-like receptors (ANPRs) with CYC/cGMP coupling as in humans. But there is no atrial natriuretic peptide detected in any sequenced placozoan genome. There are also four *Trichoplax* adenylate cyclases, which have two CYC domains (humans have nine adenylate cyclases); these are probably not involved in NO binding, and we used them as outgroups.

Unexpectedly, we discovered 12 additional guanylate cyclases with unique NIT domains^76^, which were only previously known from bacteria as nitrate and nitrite sensors^77,78^. Nitrate/nitrite sensing type domain in placozoans (NIT: PF08376) is flanked by two transmembrane domains and a C-terminal guanylate cyclase catalytic domain (AC/GC: PF00211). The same critical amino acid residues that were observed in the bacterial sequences were also present in the predicted placozoan NIT domains. (Fig. 7B). The phylogenetic reconstruction underlying the tree shows that they belong to the ANPR type/group and probably arose by lateral gene transfer into an existing ANPR type, which is established as guanylate cyclase.

To the best of our knowledge, these types of NIT containing proteins have not been previously characterized in animals. There are no detected NIT domains in the sequenced genomes of ctenophores and sponges. However, the observed NIT abundance in placozoans suggests potential sensing of nitrites and/or nitrates. This hypothesis is consistent with our present finding of the micromolar concentration of nitrites in *Trichoplax*. Because many placozoan cells (e.g., fiber cells) do contain endosymbiotic bacteria^79,80^, additional levels of intra- and intercellular NO-dependent communications are also highly likely and can be tested in future studies. Nevertheless, we do not expect that endosymbiotic bacteria produce NO and therefore cross-react with our microchemical/pharmacological assays. Although, as we showed in Fig. 2, some bacteria do contain animal-like NOS genes, the sequenced genomes of placozoan endosymbionts^79,80^ do not encode any recognizable NOS. Moreover, the estimated volume/mass occupied by endosymbionts is substantially less than these parameters in *Trichoplax* cells^79^.

Even more interesting, we found NIT-containing GCs across many bilaterian lineages, including molluscs, annelids, arthropods, priapulids, echinoderms, hemichordates and basal chordates but vertebrates lost NIT domains (Fig. 2 Supplement). Apparently, molluscs, hemichordates (*Saccoglossus*), and placozoans have one of the largest numbers of predicted NIT domain genes compared to all studied metazoans.

The model cnidarian *Nematostella* has no NIT domain, but there are NIT-containing genes in the genome of related anthozoan species, including corals. The supplementary phylogenetic tree shows that all metazoan NIT-GCs cluster together, and their NIT domains are more similar to each other than to bacterial NITs, suggesting their tracing to a common ancestor of placozoans, cnidarian, and bilaterians.

The exact function of the NIT domain in animals is yet to be elucidated, but the same architectural organization of the NIT domain^78,81^ is observed across metazoans (Fig. 7B), inferring a similar function(s). In bacteria, it has been proposed that the NIT domain regulates cellular functions in response to changes in nitrate and/or nitrite concentrations, both extracellular and intracellular^77,78^. The same possibility of nitrite/nitrate sensing might be widespread across the animal kingdom. Functional studies would be needed to carefully test this hypothesis in the future.

## Discussion

### Comparative biology of NO signaling

The phylogenetic position of Placozoa, as an early branching metazoan lineage, and the simplicity of morphological organization emphasizes the importance of *Trichoplax* as one of the critical reference species for understanding the origin and evolution of animals and their signaling mechanisms^82^, including NO-/cGMP-mediated signaling. Our combined genomic, molecular, and microchemical analyses strongly indicate the presence of functional NOSs in *Trichoplax*, which is broadly distributed across different cell populations. In contrast to other prebilaterian animals, placozoans independently evolved three different NOS genes, similar to the situation in vertebrates. This relatively recent diversification of enzymes producing gaseous free radical messenger illustrates the parallel development of complex signaling mechanisms in placozoans. It implies a much greater complexity of intercellular communications than it was anticipated before.

For Metazoans, the NOS evolution was apparently associated with the incorporation or loss of PDZ domains, its Ca-dependence, and duplication events in some lineages; but we do not exclude a possibility of the existence of pseudogene sequences as a result of gene duplication events.

The extended phylogenetic analysis with new sequences recently generated form early-branching metazoans (such as sponges and placozoans) as well as from representatives of other eukaryotic lineages strongly suggest that a complex NOS (with canonical oxygenase, flavodoxin, FAD and NAD domains) was present even in the common ancestor of all eukaryotes with apparent multiple losses of either FAD or even both FAD and Flavodoxin domains. For example, such loss occurred in the lineages leading to some parasitic eukaryotes (e.g., *Sphaeroforma*), including fungi (e.g., *Colletotrichum*). These events sometimes paralleled by the recruitment of novel domains, as we observed in *Glomerella* (Fig. 2).

However, even evolutionary distant lineages such as slime molds and some green algae maintained the evolutionary conservative NOS architecture similar to Metazoa. For the first time, we discovered the same type of multidomain NOSs both in the bacterial (*Spirosoma linguale*) and cyanobacteria (*Synechococcus* sp.) genomes, with the unusual addition of globin domains (Fig. 2). Only the oxygenase domain of NOS was found in Archaea. These findings support a deep ancestry of complex NOSs and its functions for all domains of life.

### Functions and targets of NO signaling in Placozoans

Our in situ hybridization data suggest that at least two NOSs in *Trichoplax* are constitutively expressed, with a more cell-specific expression for NOS1 and co-localization of both NOS1 (PDZ-containing) and NOS3 in the same cells. We also think than the pharmacology of NOS in *Trichoplax* requires separate attention and should be characterized in future studies, especially the Ca-dependence of different isoforms.

What are the functional roles of NO in placozoans? The physiology and cellular basis of behaviors of *Trichoplax* are poorly understood, and only a few signal molecules have been proposed for these animals so far: small secretory peptides^28,83^, glycine^84^, l-glutamate, and l-/d-aspartate^85^. But there is little doubt that NO might act as a gaseous broadly diffusible messenger controlling placozoan behaviors and immunity.

Our pilot tests (using the application of NO donor NOC9) indicated that cellular targets of NO could be both cilia and contractive cells^86^. NO can both activate and suppress cilia beating, locomotion, and contractility^13,63,87^, and therefore induce coordinated modulation of feeding behaviors and chemoreception pathways^1^. The well-described bacteriostatic properties of NO are also parts of innate immunity mechanisms broadly distributed across different species^1^. The integration of these two evolutionarily conserved functions of NO could be linked to the feeding ecology of *Trichoplax* as biofilm-eating animals.

*Trichoplax* contains intracellular bacteria, which present only in a specific and relatively small population of cells—primarily, the fiber cells^79,80^. Potentially, these bacteria could contribute to enzymatic NO production and interfere with obtained measurements of NOS related metabolites. The genomes of two bacterial endosymbionts (*Grellia incantans* (Midichloriaceae/Rickettsiales) and *Ruthmannia eludens* (Margulisbacteria) in H2 have been recently sequenced^79,80^, but they do not contain recognizable NOSs. Thus, it is highly unlikely that NO in *Trichoplax* can be produced by bacteria. Nevertheless, we do expect complex intra- and intercellular signaling between host and endosymbionts, or its contribution to innate immunity responses.

The molecular targets of NO in *Trichoplax* in different cell types can be seven soluble guanylate cyclases (sGCs) and five membrane-bound ANP-like receptors. Besides, we identified twelve cyclases with unique NIT domains. Placozoans have the largest number of predicted NIT domain genes compared to all studied metazoans. We hypothesize that in placozoans, as in bacteria, the putative NIT domain is used as nitrate/nitrite-sensing due to the high levels of nitrate/nitrites measured in *Trichoplax*. There is also a possibility that the same multidomain proteins can also bind NO itself.

In summary, although canonical functional NO-cGMP signaling could be a highly conservative feature across Metazoa, the enormous diversity of molecular components of these and related pathways in placozoans stress the cryptic complexity of these morphologically simplest animals. As one of the most versatile messengers in the animal kingdom (and in the human body), virtually all aspects of cellular and system functions might be affected by NO, as a volume transmitter, depending upon its local concentrations. The experimental determination of these localized concentrations of NO and nitrites, together with the proximity of specific molecular and cellular targets, would be critical steps to decipher the role of gaseous signaling in the integration of behaviors and other functions in *Trichoplax* and kin.

## Materials and methods

### Animals and culturing

*Trichoplax adhaerens* (H1 haplotype) and *Hoilungia hongkongensis* (H13 haplotype)^33^, 0.3–2 mm in diameter, were maintained in the laboratory culture as described elsewhere, and animals were fed on rice grains and algae^25,88^.

Direct microchemical assays of NOS metabolites such as NO_2_^−^, l-arginine, l-citrulline were performed using high-resolution capillary electrophoresis (CE) with both conductivity and laser-induced fluorescence (LIF) detectors. The principles and details of major protocols for NOS activity assay were reported^48,50,52^ with some minor modifications. We made minor adjustments to these protocols, which we briefly summarize below.

### Nitrite/nitrate microanalysis using CE with contactless conductivity

CE, coupled with a TraceDec contactless conductivity detector (Strasshof, Austria) was used for the assay of nitrite and nitrate. To reduce Cl^−^ in a sample, we used OnGuard II Ag (DIONEX Corp., Sunnyvale, CA). We used custom-built cartridges for small volume (20 μL) sample clean-up by a solid-phase extraction technique as reported^89^. In brief, 4–5 mg of the resin was backloaded in a 10 μL filter-pipette tip, and the micro-cartridge was washed with 1 mL of ultrapure water using a 3 mL disposable syringe. The pre-washed cartridge was put into a 200 μL pipette tip to avoid surface contamination during further centrifugation. Extra water remaining in the cartridge was removed by centrifugation at 1,000 rpm for 30 s. Then, the assembly was inserted into a 0.5 mL PCR tube, and a final diluted sample was loaded into the preconditioned cartridge followed by centrifugation at 1,000 rpm for 30 s, causing the sample to pass through the silver resin. To quantitate any potential sample loss, the custom-made chloride cartridge was tested for sample recovery of both nitrite and nitrate.

All experiments were conducted using a 75 cm length of 50 μm, inner diameter (I.D.) × 360 μm outer diameter (O.D.) fused silica capillary (Polymicro Technologies, AZ) with an insulated outlet conductivity cell. Arginine/borate electrolyte was used for a separation buffer with tetradecyltrimethylammonium hydroxide (TTAOH) added as an electro-osmotic flow (EOF) modifier. The modifier was prepared from tetradecyltrimethylammonium bromide (TTABr) by an OnGuard-II A cartridge (DIONEX Corp., CA) treated with 1 M NaOH. For separation steps, the capillary inner-wall was successively washed with 1 M NaOH, ultrapure water, and the separation buffer (25 mM Arg, 81 mM Boric acid, and 0.5 mM TTAOH, pH 9.0) by applying pressure (1,900 mbar) to the inlet vial. Since nitrite and nitrate concentrations were very low in diluted samples, capillary isotachophoresis (CITP), a sample stacking method, was employed. The leading solution was introduced into the capillary by pressure injection (25 mbar for 12 s), and then a sample was loaded using electrokinetic injection (− 5 kV for 12 s). The separation was performed under a stable − 15 kV voltage at 20 °C.

### Amino acids microanalysis using CE with laser-induced fluorescence detection

The CE, coupled with the ZETALIF detector (Picometrics, France), was used for the assay of amino acids^84,85^. In this work, a helium-cadmium laser (325 nm) from Melles Griot, Inc. (Omnichrome Series56, Carlsbad, CA) was used as the excitation source. Before the photomultiplier tube (PMT), the fluorescence was both wavelengths filtered and spatially filtered using a machined 3-mm pinhole. All instrumentation, counting, and high-voltage CE power supply were controlled using DAx 7.3 software.

All solutions were prepared with ultrapure Milli-Q water (Milli-Q filtration system, Millipore, Bedford, MA) to minimize the presence of impurities. Borate buffer (30 mM, pH 9.5) was used for sample preparation. All solutions were filtered using 0.2 μm filters to remove particulates. The buffers were degassed by ultrasonication for 10 min to minimize the chance of bubble formation. A 75 mM *ortho*-phthalaldehyde (OPA)/β-mercaptoethanol (β-ME) stock solution was prepared by dissolving 10 mg of OPA in 100 μL of methanol and mixing with 1 mL of 30 mM borate and 10 μL of β-ME. Stock solutions (10 mM) of amino acids were prepared by dissolving each compound in the borate buffer. OPA and β-ME were stored in a refrigerator, and fresh solutions were prepared weekly.

All experiments were conducted using a 75 cm length of 50 μm I.D. × 360 μm O.D. fused silica capillary (Polymicro Technologies, AZ). A 30 mM borate/30 mM sodium dodecyl sulfate (SDS) electrolyte (adjusted to pH 10.0 with NaOH) was used as the separation buffer for amino acid analysis. The pre-column derivatization method was used. A 1 μL of *o*-Phthalaldehyde (OPA) was incubated in a 0.5 mL PCR tube. The total volume of a sample, OPA, and internal standard inside the tube was 20 μL. For separation steps, the capillary inner-wall was successively washed with 1 M NaOH, Milli Q water, and the separation buffer by applying pressure (1,900 mbar) to the inlet vial. Then the sample was loaded using electrokinetic injection (8 kV for 12 s). The separation was performed under a stable 20 kV voltage at 20 °C.

In all CE tests, once an electropherogram was acquired, peaks were assigned based on the electrophoretic mobility of each analyte, and the assignments were confirmed by spiking corresponding standards into the sample. Five-point calibration curves (peak area vs. concentration) of analytes were constructed for quantification using standard solutions. All chemicals for buffers were obtained from Sigma-Aldrich, and standard amino acids were purchased from Fluka. Ultrapure Milli-Q was used for all solutions and sample preparations.

### NOS inhibitors’ tests

To establish that NOS enzymatic activity is responsible for producing the Arg/Cit ratio and nitrite measured in *Trichoplax*, a whole animal was incubated in one of NOS inhibitors (e.g., *N*^G^-nitro-l-arginine methyl ester (L-NAME); besides, another NOS inhibitor, L-N^6^-(1-iminoethyl)-lysine (L-NIL), showed very effective inhibition as in molluscan preparations^49^.

After the animals were isolated from the culture medium, they were placed in a 0.5 mL PCR tube and incubated with 0.5–1 mM of NOS inhibitors for 30 min at room temperature, followed by washing with artificial seawater. Then, all the water was removed, and 1 µL of Milli Q water was dropped onto the animal, and the tube was stored at − 80° C until use.

Specifically, we also performed a series of control tests to see if there were any small molecules that might interfere with peak identifications. Water, L-NAME, and L-NIL controls were first tested, and no nitrite was observed. However, chloride and nitrate ions were always present, because all NOS inhibitors contain chloride, and nitrate is a common impurity in most of the commercially used chemicals. Fresh single individuals of *Trichoplax* by itself, and *Trichoplax* incubated with NOS inhibitors were then analyzed. An effective NOS inhibitor should decrease the nitrite level compared to control tests. Absolute arginine and citrulline concentrations cannot be used as a marker of NOS activity since these are common components of cellular metabolism. However, the ratio of arginine to citrulline and the sensitivity of this ratio to NOS inhibitors is a reliable assay for the presence of functional NOS, which was validated for different species^47,48,50^.

Once an electropherogram was acquired, peaks were assigned by relative electrophoretic mobility and confirmed by spiking corresponding standards into the sample. Five-point calibration curves (peak area vs. concentration) of analytes were always constructed for quantification using standard solutions. The 3σ method was used to determine the limit of detection (LOD): LOD = 3 × σ_*blank*_/m, where *m* is the slope of the calibration line, and σ_*blank*_ is the standard deviation of the blank (usually n = 5–7). The reproducibility and accuracy of the method were evaluated by calculating the relative standard deviation (RSD) for each analyte (see details elsewhere^85^). In order to obtain the peak area, a baseline is constructed and subtracted using the derlim algorithm of DAx software version 7.3 (Van Mierlo Software Consultancy, the Netherlands). Statistical data analysis is performed by Sigma Plot software (SPSS, Inc., Richmond, CA). All results were expressed as mean and standard deviation from multiple samples, where a control group was compared with NOS inhibitor(s) treated samples using paired t-test.

### Comparative bioinformatic analyses

We used the data from the sequenced genomes of two sequenced placozoan species^33,90^, and our additional sequencing data are presented in the supplement 1. The search for possible homologs and computational annotation of predicted gene functions was performed using sequence similarity methods (BLAST/DELTA BLAST) algorithm and protein domain detection (Pfam and SMART, https://smart.embl-heidelberg.de/) as described elsewhere^91,92^. In one case, *Nematostella*, NOS gene is a part of the existing assembly with two contigs, which were not linked (https://metazoa.ensembl.org/Nematostella_vectensis/Info/Index).

Protein sequences were aligned in MUSCLE^93^. Phylogenetic trees were inferred using Maximum Likelihood algorithm implemented in IQTREE webserver https://iqtree.cibiv.univie.ac.at/^94^,. Tree robustness was tested with 2,000 replicates of ultrafast bootstrap^95,96^.

To test for positive and negative selection, the following algorithms were used: codon-based Z-test and Fischer’s exact test implemented in MEGA X^97–99^, and ABSREL, BUSTED, FUBAR and MEME in HyPhy package^100–104^. Evolutionary distances were calculated in MEGA X under the Poisson method and gamma-distributed rates across sites.

All sequences and accession information is presented in the Supplementary Dataset (excel table).

*Fixative-resistant NADPH-diaphorase activity* has been widely used as a histochemical reporter of NOS in both vertebrates and invertebrates^50,53–56^. Thus, we used this approach to screen for putative NOS activity in *Trichoplax* and *Hoilungia*. All methodological details of the protocol have been described earlier^58,59,61,63^. Briefly, we used 45 and 90 min fixation in 4% freshly made paraformaldehyde solution made using the filtered seawater. Then fixed placozoans were washed three times (10 min each) in 0.5 M Tris–HCl (pH 8.0) and placed in the staining solution (1 mM β-NADPH, 0.5 mM nitro blue tetrazolium chloride, 0.05–0.1% Triton X-100 in 0.5 M TrisHCl) and incubated, in the dark, over a period of 1–4 h at room temperature. The preparations were washed for 10–15 min in 0.5 M Tris–HCl and viewed under a microscope as we performed for the cnidarian *Aglantha*^13^. Preparations could be post-fixed for 20–60 min in 4% paraformaldehyde in methanol followed by dehydration in 100% ethanol (two times for 10–15 min). All animals were checked for any possible contamination, washed multiple times, and carefully examined under a microscope. There were no detectable other, non-*Trichoplax*, cells in these NADPH-d reactive areas, and no contamination is noted under careful microscopic investigations. All chemicals were from Sigma.

*Single-molecule fluorescent *in situ* hybridization* (smFISH) was performed using the RNAscope multiplex fluorescent Reagent kit v2 assay (Advanced Cell Diagnostics, Inc, Bio-Techne, USA) as specified by the company protocol (https://acdbio.com/rnascope%C2%AE-multiplex-fluorescent-v2-assay). As a control, we used three probes specific for different sequences, including already identified secretory peptides^28^, which showed no-cross reactivity/labeling with NOS-specific probes. In brief, we transferred 10–15 animals to the glass slides with cavities with 2 mL fresh 0.2 µm filtered seawater, washed three times, and removed the seawater under a microscope. Next, we fixed animals using 4% paraformaldehyde in seawater for 30 min at room temperature, performed dehydration and rehydration steps with increased and decreased concentrations of ethanol (30%, 50%, 70%, 100% on PBS) at room temperature. We pretreated animals in Protease III (Sigma) for 10 min at room temperature. The rest of the protocol is reported elsewhere (Advanced Cell Diagnostics, ACD #323110 at web site https://acdbio.com/rnascope%C2%AE-multiplex-fluorescent-v2-assay). The key point in the procedure is to use tyramide signal amplification steps to detect low-abundant genes as NOSs.

For all imaging, we used fluorescent microscope Nikon Ti2 (Nikon, Japan) with a spinning disk (Crest Optics X-Light V2).

## Supplementary information

Supplementary Information 1.

Supplementary Information 2.

Supplementary Information 3.

Supplementary Information 4.
