# Supplementary material for: The diversification and lineage-specific expansion of nitric oxide signaling in Placozoa: insights in the evolution of gaseous transmission
Source: Sci Rep. 2020 Aug 3;10:13020. doi: 10.1038/s41598-020-69851-w (PMC7400543; doi:10.1038/s41598-020-69851-w)
Supplement: Supplementary file 1 — Supplementary Information 1. [file 41598_2020_69851_MOESM1_ESM.docx]

**Supplement 1** includes the alignment of NOSs; **the supplementary dataset** – the summary excel table with the list of sequences used; **supplement 2** – supporting information with phylogenetic trees.

**Supporting Information:** Two Figures and one excel table with all sequences used in the figures.

**Figure 1S.** Maximum likelihood phylogenetic tree of placozoan soluble guanylate cyclases (sGC) and two groups of related enzymes: Atrial Natriuretic Peptide-like receptors (ANPRs), some of which contain unusual NIT domains, and adenylate cyclases. Proteins from all four placozoan species used in this study are represented (see Fig 7A in the main text). The references for each particular gene with relevant GeneBank accession numbers are summarized in the supplementary dataset (Excel Table).

**Figure 2S.** Maximum likelihood phylogenetic tree of NIT domains containing guanylate cyclases in placozoans (*Trichoplax* sp. H2 only), cnidarians and bilaterians. Guanylate cyclases with NIT domains are found in most animal phyla except sponges, ctenophores, vertebrates, and urochordates. Extensive lineage-specific duplications are evident in placozoans, molluscs, and hemichordates. 66 protein sequences were trimmed down to NIT+cyclase domains and produced an alignment 689 aa long. The alignment was analyzed in IQTREE ^105^ using LG+F+R6 evolution model chosen automatically with Bayesian information criterion. Tree robustness was tested with 2000 replicates of ultrafast bootstrap. The references for each particular gene with relevant GeneBank accession numbers are summarized in the supplementary dataset (Excel Table).
