# Supplementary material for: The diversification and lineage-specific expansion of nitric oxide signaling in Placozoa: insights in the evolution of gaseous transmission
Source: Sci Rep. 2020 Aug 3;10:13020. doi: 10.1038/s41598-020-69851-w (PMC7400543; doi:10.1038/s41598-020-69851-w)
Supplement: Supplementary file 2 — Supplementary Information 2. [file 41598_2020_69851_MOESM2_ESM.pdf]

Supplement 1

Nitric oxide signaling in Placozoa: Microchemical characterization and the lineage-specific diversification

Leonid L. Moroz[1¶*], Daria Y. Romanova[2¶], Mikhail A. Nikitin[3¶], Dosung Sohn[1¶], Emilie Neveu[4], Frederique Varoqueaux[4*], Dirk Fasshauer[4], Andrea B. Kohn[1,¶],

[1]Whitney Laboratory for Marine Biosciences and Departments of Neuroscience, University of Florida, St. Augustine and Gainesville, FL, 32080, USA; [2]Institute of Higher Nervous Activity and Neurophysiology, Russian Academy of Sciences, Moscow 117485, Russia; [3]Belozersky Institute of Physico-Chemical Biology, Moscow State University, Moscow 119991, Russia; [4]Department of Fundamental Neurosciences, University of Lausanne, 1005 Lausanne, Switzerland

Supplementary Data: NOS alignments (see Fig 1 and Supplementary Dataset Excel Table)

```
                                                                           PDZ
nHomo          : MEDHMFGVQQIQPNVISVRLFKRKVGGLGFLVKERVSKPPVIISDLIRGGAAEQSGLIQAGDIILAVNGRPLVDLSYDSA :  80
eHomo          : -------------------------------------------------------------------------------- :   -
iHomo          : -------------------------------------------------------------------------------- :   -
Trichoplax_H1-1: MEELRDT--TIQVTKDNISENYGFQVKNYDAMTYCTVSHIVDNNNKNK--ANPDISELQLGDLLIAVNDISLADQSVEDM :  76
Trichoplax_H1-2: -------------------------------------------------------------------------------- :   -
Trichoplax_H1-3: -------------------------------------------------------------------------------- :   -
Hoilungia_13-2 : -------------------------------------------------------------------------------- :   -
Hoilungia_13-1 : MEELYDNGMTIEVVKDIVNGNYGFQVKKNDTMPYCSVSHIVDNHADVKNINGSDQKRLQLGDLLLAVNDISLADKSAEDI :  80

nHomo          : LEVLRGIASETHVVLILRGPEGFTTHLETTFTGDGTPKTIRVTQPLGPPTKAVDLSHQPPAGKEQPLAVDGASGPGNGPQ : 160
eHomo          : -----------------MGN-----------------------------LKSVAQEPGPPCG----------LGLGLG-- :  22
iHomo          : ----------------MACP--------------------------WKFLFKTKFHQYAMNGE---------KDINNN-- :  27
Trichoplax_H1-1: YDIIDQNSKNDMVNLTIRSKHQLLN-----------SNNHNGGENITKLNGNSRIKIKRKIGLGRDPSLDGTFESNNNQD : 145
Trichoplax_H1-2: -------------------------------------------------------------------------------- :   -
Trichoplax_H1-3: -------------------------------------------------------------------------------- :   -
Hoilungia_13-2 : -------------------------------------------------------------------------------- :   -
Hoilungia_13-1 : YEIIDKNTKNNVVNLTIRPNESNEN-----------PGQLQT-DSYRQLNGTSRAKVKRKIGLSKDPSLDNNIYENN--- : 145

nHomo          : HAYDDGQEAGSLPHANGLAPRPPGQDPAKKATRVSLQGRGENNELLKEIEPVLSLLTSGSRGVKGGAPAKAEMKDMGIQV : 240
eHomo          : -----------LGLCGKQGPATPAPEPSR--------------------------------------------------- :  40
iHomo          : -----------VEKAPCATSSPVTQDDLQYHN------------------------------------------------ :  48
Trichoplax_H1-1: HGQDSNNGKKASPIAQINIDELMASDDDAIGSQNDCPEQPVDGVNLR--------------------------------- : 192
Trichoplax_H1-2: -------------------------------------------------------------------------------- :   -
Trichoplax_H1-3: -------------------------------------------------------------------------------- :   -
Hoilungia_13-2 : -------------------------------------------------------------------------------- :   -
Hoilungia_13-1 : TGHQDVTTKNTLPIGQININELMASDDDGDENNLDSSQQPIDNVKLR--------------------------------- : 192

nHomo          : DRDLDGKSHKPLPLGVENDRVFNDLWGKGNVPVVLNNPYSEKEQPPTSGKQSPTKNGSPSKCP----------------- : 303
eHomo          : ------------------------------APASLLPPAPEHSPPSSPLTQ-------PPEGP----------------- :  66
iHomo          : -----------------------------LSKQQNESPQPLVETGKKSPESLVKLDATPLSSP----------------- :  82
Trichoplax_H1-1: -----------VKKNSEVDKIPAKKWTKEAVLEDLTTDKSKINKLLYTRSRRCQSDGSQGSQKHLTKLLINGKEIPGAIA : 261
Trichoplax_H1-2: -----------------------------------MDGKSQEWLWPSPATVSSPNSDSNGP------------------- :  26
Trichoplax_H1-3: -----------------------------------MDSQS-------------AVPNLEGQ------------------- :  13
Hoilungia_13-2 : -----------------------------------MDSQSQEWEWPSPMSILT-KADSNGP------------------- :  25
Hoilungia_13-1 : -----------VKQHSNIDKITPKQSTKQVALEDMITDKSKINKLLYTRSRRCQNDSIQVNQKHQTKITINGKEIPGSIP : 261

                           HPL            --Zn--  Pterin
nHomo          : --------RFLKVKNWETEVVLTDTLHLKSTLETGCTEYICMGSIMHPSQHARRPE-DVRTKGQLFPLAKEFIDQYYSSI : 374
eHomo          : --------KFPRVKNWEVGSITYDTLSAQAQQDGPCTPRRCLGSLVFPRKLQGRPSPGPPAPEQLLSQARDFINQYYSSI : 138
iHomo          : --------RHVRIKNWGSGMTFQDTLHHKAKGILTCRSKSCLGSIMTPKSLTRGPRDKPTPPDELLPQAIEFVNQYYGSF : 154
Trichoplax_H1-1: EED-TSKNGFIRLKNWSNSSYLHDTLHKKAKCPMTCGSQVCYGSSMNFLPEQKPEYTTESYRQYIIDQAVNFIREYYLNL : 340
Trichoplax_H1-2: --------SFIRLQNLLTGEQNTDTFHQQAATTPGCTFRRCLG-SLMQVPSQTKRHNGLKPKEVVIKEAKDFLMEYYASL :  97
Trichoplax_H1-3: --------TFMRLQNLITTEQTTDTFHQRATTIPSCTGKRCLG-SLMQIPEKIKRENGLKPKELVIKEATEFLQEYYASI :  84
Hoilungia_13-2 : --------SFIRLQNLLTNGQNTDTFHQQAITTQSCTSKRCLG-SLMQIPNQTKRHNGLKPKELVIKEAKEFLREYYASL :  96
Hoilungia_13-1 : EEDDASKNNFIRLKNWSNNSYLHDTLHTKAKCPMTCASQYCYGSSMNYIPEKKPECNTERNRQYVIDQAVSFIREYYHHL : 341

                                                   HEME
nHomo          : KRFGS-------KAHMERLEEVNKEIDTTSTYQLKDTELIYGAKHAWRNASRCVGRIQWSKLQVFDARDCTTAHGMFNYI : 447
eHomo          : KRSGS-------QAHEQRLQEVEAEVAATGTYQLRESELVFGAKQAWRNAPRCVGRIQWGKLQVFDARDCRSAQEMFTYI : 211
iHomo          : KEAKI-------EEHLARVEAVTKEIETTGTYQLTGDELIFATKQAWRNAPRCIGRIQWSNLQVFDARSCSTAREMFEHI : 227
Trichoplax_H1-1: RNENPNSNIDFEKEELARLEQIIEDVEQTGFYEQTYSELVFGAKLAWRNATRCIGRIQWSRLKVFDARHVKTTDEMFQAI : 420
Trichoplax_H1-2: RKENT-------EAHLKRQADVIDEIQAKGLYELTEQELTYGCRLAWRNASRCIGRIQWGNLHVFDARDAVTPEDMFNAI : 170
Trichoplax_H1-3: RKEGS-------EAHLKRKADVIEEIQAKGLYDLTEQELTYGARLSWRNASRCIGRIQWSNLHIFDARDAVTATDMYDAI : 157
Hoilungia_13-2 : RKENT-------EAHLKRETDVIDQIQTKRIYDLTEQELTYGARLAWRNASRCIGRIQWANLHLFDARDAVTPEDMYNVI : 169
Hoilungia_13-1 : MSENENPDIDYEKEELARLEQIIEDVENTGYYELTYNEIVFGAKLAWRNATRCIGRIQWSRLKVFDARHVKTTDEMFQAI : 421

                                   PF02898: NO_synthase
nHomo          : CNHVKYATNKGNLRSAITIFPQR-TDGKHDFRVWNSQLIRYAGYKQPDGSTLGDPANVQFTEICIQQGWKPPRGRFDVLP : 526
eHomo          : CNHIKYATNRGNLRSAITVFPQR-CPGRGDFRIWNSQLVRYAGYRQQDGSVRGDPANVEITELCIQHGWTPGNGRFDVLP : 290
iHomo          : CRHVRYSTNNGNIRSAITVFPQR-SDGKHDFRVWN-------------------------AQLCIDLGWKP--------- : 272
Trichoplax_H1-1: CEHLKYATNNGNLRSVLTIFAPRNGSKQNDFRIWNAQLIRYAGYKMDDGSIIGDRATVEFTELCLSFGWRGSGGRFDVLP : 500
Trichoplax_H1-2: TDHVKYATNKGNIS-------------MHDFRVWNVQLIRYAGYEQADGSVIGDPSNIEFTKICQSYGWRGNGGMFDVLP : 237
Trichoplax_H1-3: ISHLKYGTNNGNLRSAMTIFPPR-ANDSHDFRVWNIQLVRYAGYEQPDGSVIGDPANVEFTKICESYGWKGKGGMFDVLP : 236
Hoilungia_13-2 : IDHLKYATNKGNLRSAITIFPPR-SDGLHDFRVWNVQLIRYAGYEQPDGSVIGDPANVEFTQICQSYGWRGKGGMFDVLP : 248
Hoilungia_13-1 : CEHLKYATNNGNLRSVITIFAPRNGTKQNDFRIWNAQLIRYAGYKMDDGSIVGDRATVEFTELCLSLGWRGSGGRFDVLP : 501

                                                           Cav
nHomo          : LLLQANGNDPELFQIPPELVLEVPIRHPKFEWFKDLGLKWYGLPAVSNMLLEIGGLEFSACPFSGWYMGTEIGVRDYCDN : 606
eHomo          : LLLQAPDDPPELFLLPPELVLEVPLEHPTLEWFAALGLRWYALPAVSNMLLEIGGLEFPAAPFSGWYMSTEIGTRNLCDP : 370
iHomo          : -----NGRDPELFEIPPDLVLEVAMEHPKYEWFRELELKWYALPAVANMLLEVGGLEFPGCPFNGWYMGTEIGVRDFCDV : 347
Trichoplax_H1-1: LILQANGGEPQFYEIPPELVLEVCLRHPKYPWFENLGLKWYALPAVANMMFDVGGIEFPCAPFNGWYMGTEIGSRDLGDT : 580
Trichoplax_H1-2: LLLQANGTEPHLFEIPEELILEVPISHPEYDWFEELKLRWYALPAVSCLLLDIGGVEFPACPFNGWYMVTEIGARDLGDT : 317
Trichoplax_H1-3: LLLQANGEDPELFEIPSDLVLEVPISHPEYEWFEELKLRWYALPAVSCLLLDIGGVEFPACPFNGWYMVTEIGARDFGDE : 316
Hoilungia_13-2 : LLLQANGSEPQIFEIPPELVLEVPISHPEYEWFEKLCLQWYALPAVSCLLLDIGGIEFPACPFNGWYMVTEIG-RDFGDI : 327
Hoilungia_13-1 : LLLQANGGEPQVYDIPSELVLEIQIRHPKYPWFENLGLKWYALPAVSNMMFDVGGIEFPCAPFNGWYMGTEIGSRDLGDV : 581

                                   PF02898: NO_synthase
nHomo          : SRYNILEEVAKKMNLDMRKTSSLWKDQALVEINIAVLYSFQSDKVTIVDHHSATESFIKHMENEYRCRGGCPADWVWIVP : 686
eHomo          : HRYNILEDVAVCMDLDTRTTSSLWKDKAAVEINVAVLHSYQLAKVTIVDHHAATASFMKHLENEQKARGGCPADWAWIVP : 450
iHomo          : QRYNILEEVGRRMGLETHKLASLWKDQAVVEINIAVLHSFQKQNVTIMDHHSAAESFMKYMQNEYRSRGGCPADWIWLVP : 427
Trichoplax_H1-1: YRYNVCETVGQHMGLDTKNNSSLWKDKALVELNIAVLYSFQEDNVTIVDHHSVSESFMNHLEHETSIRGGCPSDWVWLVP : 660
```

```
Trichoplax_H1-2 : NRYNLCKPVAQYLKLDTRSETTLWRDKAIVELNRAVMHSFLKHKVTLVDHHTASNTFMQHLDNEQKLRGGCPADWVWIVP :  397
Trichoplax_H1-3 : NRYNLCKPVAQRMKLDTRTETTLWRDKAIVEINCAVMHSFLKHGVTLVDHHSTSNSFMQHLQNEQKLRGGCPADWVWVVP :  396
Hoilungia_13-2  : NRYNMCKPVAQYLKLDTRSETTLWRDKAIVELNRAVMYSFLKHKVTMVDHHTASNSFIQHLQNEQKLRGGCPADWVWIVP :  407
Hoilungia_13-1  : YRYNLCETVGQHMGLDTKNNSSLWKDKALVELNVAVLYSFQEDNVTIVDHHSVSESFMNHLQHETSTRGGCPSDWVWLVP :  661

                                                                          CaM
nHomo           : PMSGSITPVFHQEMLNYRLTPSFEYQPDPWNTHVWKGTNGTPTKRRAIGFKKLAEAVKFSAKLMGQAMAKRVKATILYAT :  766
eHomo           : PISGSLTPVFHQEMVNYFLSPAFRYQPDPWKGSAAKGTGITRKKT----FKEVANAVKISASLMGTVMAKRVKATILYGS :  526
iHomo           : PMSGSITPVFHQEMLNYVLSPFYYYQVEAWKTHVWQDEKRRPKRR-EIPLKVLVKAVLFACMLMRKTMASRVRVTILFAT :  506
Trichoplax_H1-1 : PLSGSASEVYHQELLSYRLKPSFEYQPDPAIDYYKR--KRKRRIK----FKNAGNAILFAHYLIKNTLAKRPKVTILYAT :  734
Trichoplax_H1-2 : PISGSATKVYHQEMLNYKIKPSFEYQEDPWKIH-----TKGSQRK----FKDIAKLVVVSTDLMGKVLAKRIRATILYAT :  468
Trichoplax_H1-3 : PISGSATKVFHQEMLNYKIKPSLEYQVDAWKIHRRKTPIKTKEGK----FKDIAKMVVRLSTELMAKALAKRIKATILYST :  472
Hoilungia_13-2  : PISGSMTKVYHQEMLNYKIKPCFEYQEDPWKIP-----IKGTQRK----FKDIAKLVVVSTDLMGKALAKRIRATILYAT :  478
Hoilungia_13-1  : PMSGSASEVYHQELLSYRLKPSFEYQPDPSIDYYKR--KRKRKIK----FKDAGNAILFAHYLIKNTLAKRPKVTILFAT :  735

                                          PF00258: Flavodoxin_1
nHomo           : ETGKSQAYAKTLCEIFKHAFDAKVMSMEEYDIVHLEHETLVLVVTSTFGNGDPPENGEKFGCALMEMRHPNSVQ---EER :  843
eHomo           : ETGRAQSYAQQLGRLFRKAFDPRVLCMDEYDVVSLEHETLVLVVTSTFGNGDPPENGESFAAALMEMSGPYNSSPRPEQH :  606
iHomo           : ETGKSEALAWDLGALFSCAFNPKVVCMDKYRLSCLEEERLLLVVTSTFGNGDCPGNGEKLKKSLFMLKELNN-------- :  578
Trichoplax_H1-1 : ETGKSEYYAKIVTNIFSYGFDVKLVCMKDYDPSNLSQEKLLLIVTSTFGNGDPPDNGVKFGSYLYQRCNPRAKVR----- :  809
Trichoplax_H1-2 : ETGTSERYAKMLKKLLDIKFDAKVVCMADYDFSNLDYEKLVFIVASTFGNAEAPHNGEEFGRRLHFRAHPID-------- :  540
Trichoplax_H1-3 : ETGTSERYGKMLKKLLDLAFDAKIYCLENYDFNNLDYENLVFVVTSTFGNADPPHNGEEFDKHLRHR------------- :  539
Hoilungia_13-2  : ETGTSERYAKMLKKLLDIKFDAKVICMEDYDFSNLDYEKLVFIVTSTFGNAEAPHNGEEFGRRLHYRSYPVDYQMD---- :  554
Hoilungia_13-1  : ETGKSEYYAKIVTHVFSYGFDVKLICMKDYDPSNLPQEKLMLIVTSTFGNGDPPENGVKFGNYLYQRCNPRAKVR----- :  810

                      AIL                                          FMN
nHomo           : KSYKVRFNSVSSYSDSQKSSGDGPDLRDNFESAGPLANVRFSVFGLGSRAY-PHFCAFGHAVDTLLEELGGERILKMREG :  922
eHomo           : KSYKIRFNSISCSDPLVSSWRRKRKESSNTDSAGALGTLRFCVFGLGSRAY-PHFCAFARAVDTRLEELGGERLLQLGQG :  685
iHomo           : ------------------------------------KFRYAVFGLGSSMY-PRFCAFAHDIDQKLSHLGASQLTPMGEG :  620
Trichoplax_H1-1 : --------ENRRSIHLPRGSNKPM---LSRIASSNYSNMKYAVFGLGSRAY-PHFCAFARSVDRLLCHMGAERIHDIGEG :  877
Trichoplax_H1-2 : -----LNASRTGSLHLPRRKSKLRANETEDRTSSFMINVKYSVFALGSRAYGENFCAFGRSLDTLLRDFGGERICPLGEG :  615
Trichoplax_H1-3 : -------------LHSYHVQNQEEDYNALNDYGTRILNVKYSVFALGSRVYGDNFCAFGRFIDASLHKMGAERICPLGEG :  606
Hoilungia_13-2  : -----LNASRTGSLHLPRRKTKIRSDEPDDRASTSMRNLKYSVFALGSRAYGENFCAFGRSLDSLLHNFGGERIYPLGEG :  629
Hoilungia_13-1  : --------ENRRSIHLPRSSNRPP---ISRITSSNYSNTKYAVFGLGSRAY-PHFCAFARSVDRLLGHMGAERIHAIGEG :  878

nHomo           : DELCGQEEAFRTWAKKVFKAACDVFCVGDDVNIEKANNSLISNDRSWKRNKFRLTFVA---EAPELTQGLSNVHKKRVSA :  999
eHomo           : DELCGQEEAFRGWAQAAFQAACETFCVGEDA--KAAARDIFSPKRSWKRQRYRLSAQA---EGLQLLPGLIHVHRRKMFQ :  760
iHomo           : DELSGQEDAFRSWAVQTFKAACETFDVRGKQ--HIQIPKLYTSNVTWDPHHYRLVQDS---QPLDLSKALSSMHAKNVFT :  695
Trichoplax_H1-1 : DELFGQEDSFRKWTEEIFHISCESFHLKSIDKKDALS-TLYKNSNHWYPGKFRMQEESLDIEKTDICIGLSKVHGKEVIS :  956
Trichoplax_H1-2 : DELCGQEESFRSWAGKAFRAACETFCIDA-VDHSDAT-TSLTTIPSWSENNFRLVFDKKKTAAG-LLEALSELHSTHIEP :  692
Trichoplax_H1-3 : DELCGQEESFKIWADNALRTACNTFSIDLNSDFKTTN-TLLTTVPAWSESNYRLVHKEKKPIKP-LLEALSKIHSKKIQS :  684
Hoilungia_13-2  : DELCGQEESFRSWAGEAFRAACDTFCIDT-VDDSDAV-VSLTTIPQWSEKNFRLVFNKKKTVVG-FMEALSKIHSTQVQP :  706
Hoilungia_13-1  : DELFGQEDSFRKWTEDVFHISCDLFHLKSINKKDALS-TLYKNSNHWYPGKFRIQMESSDIEKPNICIGLSKVHGKEVIP :  957

                                                FAD-PPi
nHomo           : ARLLSRQNLQSPKSSRSTIFVRLHTNGSQELQYQPGDHLGVFPGNHEDLVNALIERLEDAPPVNQMVKVELLEERNTALG : 1079
eHomo           : ATIRSVENLQSSKSTRATILVRLDTGGQEGLQYQPGDHIGVCPPNRPGLVEALLSRVEDPPAPTEPVAVEQLE-KGSPGG :  839
iHomo           : MRLKSRQNLQSPTSSRATILVELSCEDGQGLNYLPGEHLGVCPGNQPALVQGILERVVDGPTPHQTVRLEALDESGSY-- :  773
Trichoplax_H1-1 : GKLISRQNLQSTSSDRSTILVRIGTKGKKSLSFHPGDHIAIFPANHPRLVQRLINALHDAPDPDQPIKIEYCQEK----A : 1032
Trichoplax_H1-2 : LTVLSVTNLQSSSSLRSTVLVKLKKASESNLCYFPGDHLSVFPRNDPEIVDQLLRKLADKESSDITTQLEYLSN------ :  766
Trichoplax_H1-3 : ATVISVTSLQSTISLHYTILVKLDKGNNSSLNYAAGDHLSIFPGNDPEIVDALLTKLTDGQSPDTPIQMEYLNN------ :  758
Hoilungia_13-2  : LTVISVTNLQSPSSQRSTILVKLSMDNTSNLCYLPGDHLSVFPCNDPEMVSQLLDKLADKESSDIAAQLEYLNS------ :  780
Hoilungia_13-1  : GKLISRENLQSASSDRSTILVRIDTRGKKSLSFHPGDHIAIFPANHPRLVQRLIDALHNAPNPDLPIKIQYCQEK----S : 1033

                                          PF00667: FAD_binding_1
nHomo           : VISNWTDELRLPP-CTIFQAFKYYLDITTPPTPLQLQQFASLATSEKEKQRLLVLSKGLQEYEEWKWGKNPTIVEVLEEF : 1158
eHomo           : PPPGWVRDPRLPP-CTLRQALTFFLDITSPPSPQLLRLLSTLAEEPREQQELEALSQDPRRYEEWKWFRCPTLLEVLEQF :  918
iHomo           : ----WVSDKRLPP-CSLSQALTYFLDITTPPTQLLLQKLAQVATEEPERQRLEALCQ-PSEYSKWKFTNSPTFLEVLEEF :  847
Trichoplax_H1-1 : GQKIWQVFDRLPLPCTLRDAFTYYLDITTPPTPQMLQHLATEAARDTDKQRLELLGRGSQKYEDWKFEKCPHIIEVIEEF : 1112
Trichoplax_H1-2 : -EGIWTVDTHMPATFTLREAFMRFIDITSPPTPQFLHCLTSLAMDQNDRNKLAELAKGSKEYEDWKFWKNPGLIDVIEEF :  845
Trichoplax_H1-3 : -EKNWTVDNHIPPIFTLREAFSRFVDISTPPTPQFLQYLVNQAKDEKEKSHLEELAKGQDEYEDWKFYENPNLLDVIDKF :  837
Hoilungia_13-2  : -EGNWTVDTYIPTGFTLREAFLRFVDITSPPTPQFLQYLINLAADKDDRDRLAELAKGSKEYEDWKFWRNPGLIDLIEEF :  859
Hoilungia_13-1  : GQKIWQVFDRLPLPCTLRDAFTYYLDITTPPTPQMLQHLATEASRDTDKQRLELLGRGSQKYEDWKFEKCPHIIEVIEEF : 1113

                            FAD-Iso
nHomo           : PSIQMPATLLLTQLSLLQPRYYSISSSPDMYPDEVHLTVAIVSYRTRDGEGPIHHGVCSSWLNRIQADELVPCFVRGAPS : 1238
eHomo           : PSVALPAPLLLTQLPLLQPRYYSVSSAPSTHPGEIHLTVAVLAYRTQDGLGPLHYGVCSTWLSQLKPGDPVPCFIRGAPS :  998
iHomo           : PSLRVSAGFLLSQLPILKPRFYSISSSRDHTPTEIHLTVAVVTYHTRDGQGPLHHGVCSTWLNSLKPQDPVPCFVRNASG :  927
Trichoplax_H1-1 : PSLKVSPLLLLIQLPLLQQRYYSISSSPKLHKNEIHCTVAVVSYRTSGGLGVKHEGVCSNWLTRLEAGATIPCFVRPDAA : 1192
Trichoplax_H1-2 : PSVRLPATLLLKLPRLKQRYYSISSSMAMTPDEIHITVSVVAYHTKS--GKLRRGICSNWLNSLKEGESVPCFIRAAHG :  923
Trichoplax_H1-3 : ESLQIQSTLLSKLPRLKQRYYSISSSMAVFPNEIHITVGVVQYYTKS--GKLCRGVCSNWLNSLKEGATIHCFTKAAPS :  915
Hoilungia_13-2  : SSLRLPATLLSKLPRLKQRYYSISSSMAMVSDEIHITVSVVTYYTKA--GKLRRGICSNWLNSLKKGDVVPCFIRAANG :  937
Hoilungia_13-1  : PSLKVSPLLLLIQLPLLQQRYYSISSSPKIYKNEIHCTVAVVSYRTSGGLGVKHEGVCSNWLTRLEAGSIIPCFVRPDAA : 1193

                              NADPH-Ribose
nHomo           : FHLPRNPQVPCILVGPGTGIAPFRSFWQQRQFDIQH-----------------------------KGMNP---------- : 1279
eHomo           : FRLPPDPSLPCILVGPGTGIAPFRGFWQERLHDIES-----------------------------KGLQP---------- : 1039
iHomo           : FHLPEDPSHPCILIGPGTGIAPFRSFWQQRLHDSQH-----------------------------KGVRG---------- :  968
Trichoplax_H1-1 : FHMPEDVSVPIILIGPGTGIAPFRSFWQQRFYDMNLSTHRPVEQDIAISTHSKFAANIEKKRSHLRRTQSDASDYPPTGT : 1272
Trichoplax_H1-2 : FRMPPDQSVPIIMIGPGTGIAPFRSFWQQRLADKNR----------------EAAFPVTVTKDYEDTEIPTGIDTPPLTP :  987
Trichoplax_H1-3 : FQMPSNNSIPIIMVGPGTGIAPFRSFWQQRAIECK------------------AATAGSDAQKEEDNSSNN--------- :  968
Hoilungia_13-2  : FHLPEDHSTPVIMIGPGTGIAPFRSFWQQRMSDKNK----------------EANTIFSDTKEHENAEISNGNDTPPLTP : 1001
Hoilungia_13-1  : FHMPEDWSVPIILIGPGTGIAPFRSFWQQRYYDMKLSPHRPVEQDIAVPKHSKFTASLDRKRSHLRRTQSDAADYPSPRA : 1273
```

```
                                                PF00175: NAD_binding_1

                      --------------------------------------------------------------------------------
nHomo               : --------------------------CPMVLVFGCRQSKIDHIYREETLQAKNKGVFRELYTAYSREPDKPKKYVQDILQE : 1334
eHomo               : --------------------------TPMTLVFGCRCSQLDHLYRDEVQNAQQRGVFGRVLTAFSREPDNPKTYVQDILRT : 1094
iHomo               : --------------------------GRMTLVFGCRRPDEDHIYQEEMLEMAQKGVLHAVHTAYSRLPGKPKVYVQDILRQ : 1023
Trichoplax_H1-1     : RSKDRGETDVSGKSKNHCSVAKSVFGEMILYFGCRNSKTDDIYKDELRKALTDHVLTHVQTAYSRQEHKPKQYVQDLIKK : 1352
Trichoplax_H1-2     : TENEDLETR----------------FGKMTLFFGCRESTKDDIYMDELKDCLTNGVLDVVHKAYSREPGQQKRYVQDLMKD : 1052
Trichoplax_H1-3     : -------------------------FGDMVLYFGCRTPVKDDIYREELEDCVNKKVIKEVYKAYSRAPDEPKQYVQDILSQ : 1024
Hoilungia_13-2      : TGNEGLATR----------------FGKMILFFGCRESTKDDIYKKEIEECLNNGVLDEVYKAYSRELNQPKQYVQDLMKE : 1066
Hoilungia_13-1      : RSKDREESDNIESS--NSSVVKGVFGEMILYFGCRNAKIDDIYKDDLRKALTDHVLTHVQTAYSRQEHKAKQYVQDLIKK : 1351

                                 ______NADPH-A_____                                 _NADPH_
                      -----------------------------
nHomo               : QLAESVYRALKEQGGHIYVCGDVTMAADVLKAIQRIMTQQGKLSAEDAGVFISRMRDDNRYHEDIFGVTLRTYEVTNRLR : 1414
eHomo               : ELAAEVHRVLCLERGHMFVCGDVTMATNVLQTVQRILATEGDMELDEAGDVIGVLRDQQRYHEDIFGLTLRTQEVTSRIR : 1174
iHomo               : QLASEVLRVLHKEPGHLYVCGDVRMARDVAHTLKQLVAAKLKLNEEQVEDYFFQLKSQKRYHEDIFGAVFPYEAKKDRVA : 1103
Trichoplax_H1-1     : D-AFELCKLILDHGAHIYVCGDVSMSADVGRTIQNVLEDYGAMSSTESQECITRLRDSGRYHEDIFGVTLRTFEVTTNRR : 1431
Trichoplax_H1-2     : H-GGDLLHTILDEDGHIYVCGDVSMAADVSRTIQNLLIDYGLFDQEDAIEYVKTMKERGRYHEDIFGITLRTGEVTERIR : 1131
Trichoplax_H1-3     : N-GNEVLAMLLDDDGHIYVCGDVSMAAGVTRAIQNILIDYGQFSQEDALEMTKNLKKNGRYHEDIFGLTLRTAEVTEKNR : 1103
Hoilungia_13-2      : H-GGDLLHMILDGDGHIYVCGDVSMAADVFRTLQNLLIDYGLFNQEDAIEYVKNMKETGRYHEDIFGITLRTGEVTERIR : 1145
Hoilungia_13-1      : D-AFELCKLILDHGAHIYVCGDVSMSADVGRTIQNVLEDYGAMSNTESQECMTRLRDSGRYHEDIFGVTLRTFEVTTTRR : 1430


nHomo               : SESIAFIEESKKDTDEGFQLLTGPSCPAGCKFCKRGQTLLNLSSGTPCGPRSASCPCRCALVSLLGLLAPQWFPRPSWVY : 1494
eHomo               : TQSFSLQERQLRG------------------------------------------------------------------- : 1187
iHomo               : VQPSSLEMSAL--------------------------------------------------------------------- : 1114
Trichoplax_H1-1     : KAAQRAWILVSGDDKTVKASASGSSIIPRIFPKAGGGVSAGSPKLRHKVRSRRARHSPTVTVLLNPDEQQQLFNSNTET- : 1510
Trichoplax_H1-2     : KAAQRAWVMIRKHEFRKSNKGDGNGIAN----------LVDKVRARQKLDTSPLERGISVDVVMRTEDEVPEQKSQSLSR : 1201
Trichoplax_H1-3     : RASQRTWTMISKRLKGETGKG--TEMAN----------LIQKLKLRKKSGSDVRGRYM---------------------- : 1149
Hoilungia_13-2      : KAAQRAWVMIRKYNFGKSHKANDNRVAD----------LVNKVQANQTLDKFLVDRGISVDVVMRTEDEIPEQKPRSFSR : 1215
Hoilungia_13-1      : KAAQRAWILVSGDDKTVKASASGSSIIPRIFPKAGGGVSAGSPKLRHKFRARRGRHSPTVTVLLNPDEQQQLFNNNTEAN : 1510


nHomo               : SLSFPAAMQCFSNLQWLLQNSVPTPSLADKGNSRVHETTGTWPSLWGFFSLGFPWKGCRN : 1554
eHomo               : ------------AVPWAFD-----PPGSDTNSP--------------------------- : 1203
iHomo               : ------------------------------------------------------------ :    -
Trichoplax_H1-1     : QTSTESNFKRLLERRHSVHAAKPDECDTEDSDDEF------------------------- : 1545
Trichoplax_H1-2     : IFSMPGSDGPTMSISASFSGEQLNISSIESYEDE-------------------------- : 1235
Trichoplax_H1-3     : ------------------------------------------------------------ :    -
Hoilungia_13-2      : VFSMPGSDGPTMSISTSFSGEHLTISSIESQEDQQ------------------------- : 1250
Hoilungia_13-1      : TTTAESKFKRLLERRHSVHAAKPDDMDTEDSDDCL------------------------- : 1545
```

Abbreviations:

PDZ domain
HPL: N-terminal hairpin loop region
Zn: Zinc binding site
Pterin-binding site
Heme: any compound of iron complexed in a porphyrin (tetrapyrrole) ring
Cav: Caveolin binding consensus sequence
CaM: Calmodulin binding domain
AIL: Auto-Inhibitory loop
FMN: Flavodoxin binding domain
FAD-PPi: Flavin adenine dinucleotide pyrophosphate binding site
FAD-Iso: Flavin adenine dinucleotide isoalloxazine binding site
NADPH-Ribose: Nicotinamide adenine dinucleotide phosphate-oxidase ribose binding site
NADPH-A: Nicotinamide adenine dinucleotide phosphate-oxidase adenine binding site

Of note, *Trichoplax* NOS1 (Trichoplax_H1-1) and NOS3 are (Trichoplax_H1-3) are constitutively expressed, as shown in Fig. 6 (main text) and from reported transcriptomes (Sebe-Pedros et al. 2018; Moroz, 2018).
