# Supplementary material for: The diversification and lineage-specific expansion of nitric oxide signaling in Placozoa: insights in the evolution of gaseous transmission
Source: Sci Rep. 2020 Aug 3;10:13020. doi: 10.1038/s41598-020-69851-w (PMC7400543; doi:10.1038/s41598-020-69851-w)
Supplement: Supplementary file 3 — Supplementary Information 3. [file 41598_2020_69851_MOESM3_ESM.pdf]

Supplement 2

Nitric oxide signaling in Placozoa: Microchemical characterization and the lineage-specific diversification

Leonid L. Moroz[1¶*], Daria Y. Romanova[2¶], Mikhail A. Nikitin[3¶], Dosung Sohn[1¶], Emilie Neveu[4], Frederique Varoqueaux[4*], Dirk Fasshauer[4], Andrea B. Kohn[1¶],

[1]Whitney Laboratory for Marine Bioscience and Departments of Neuroscience, University of Florida, St. Augustine and Gainesville, FL, 32080, USA; [2]Institute of Higher Nervous Activity and Neurophysiology, Russian Academy of Sciences, Moscow 117485, Russia; [3]Belozersky Institute of Physico-Chemical Biology, Moscow State University, Moscow 119991, Russia; [4]Department of Fundamental Neurosciences, University of Lausanne, 1005 Lausanne, Switzerland

Two supplementary figures: Phylogenetic Trees

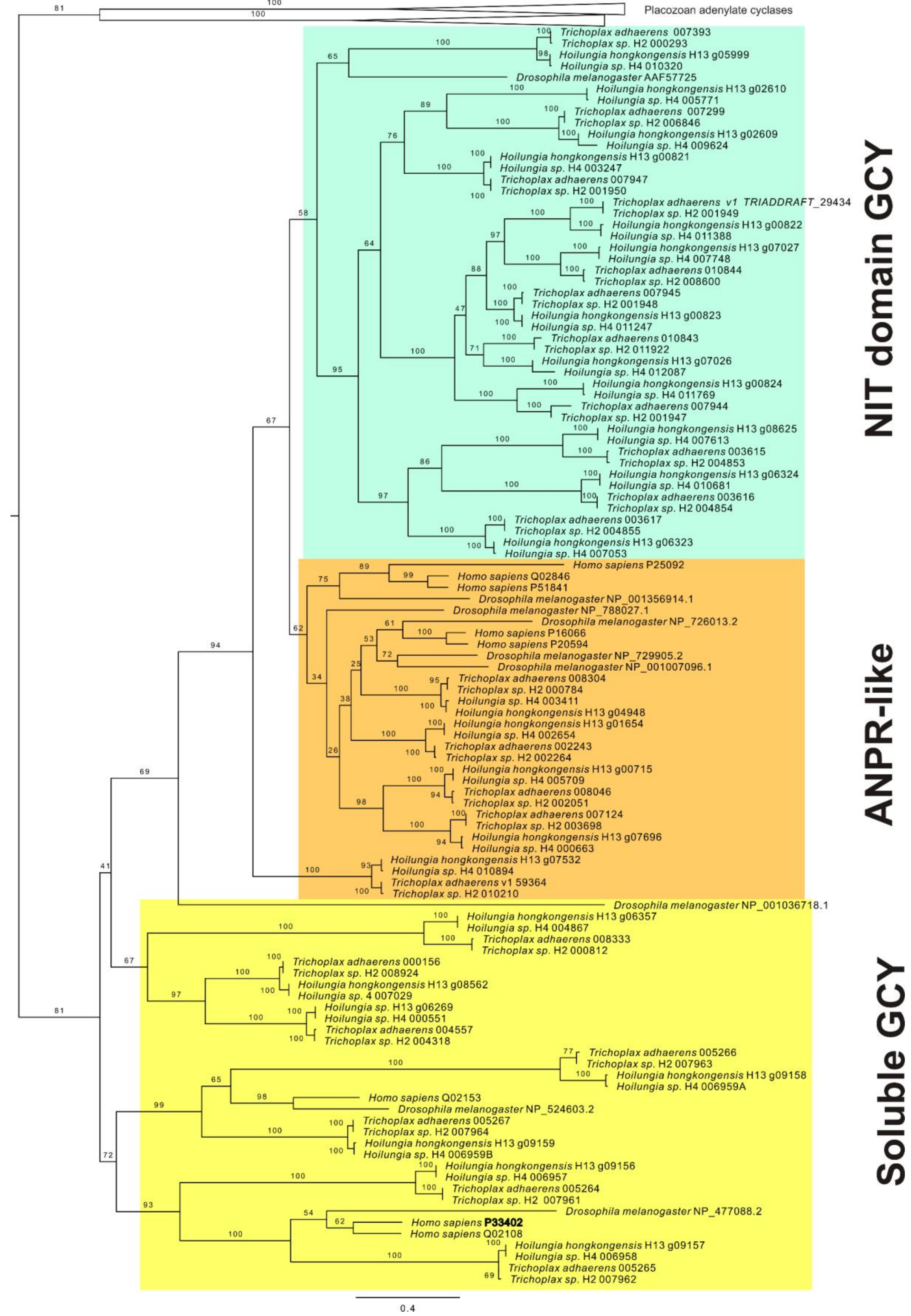

Figure 1S. Maximum likelihood phylogenetic tree of placozoan soluble guanylate cyclases (sGC) and two groups of related enzymes: Atrial Natriuretic Peptide-like receptors (ANPRs), some of which contain unusual NIT domains, and adenylate cyclases. Proteins from all four placozoan species used in this study are represented (see Fig 7A in the main text).

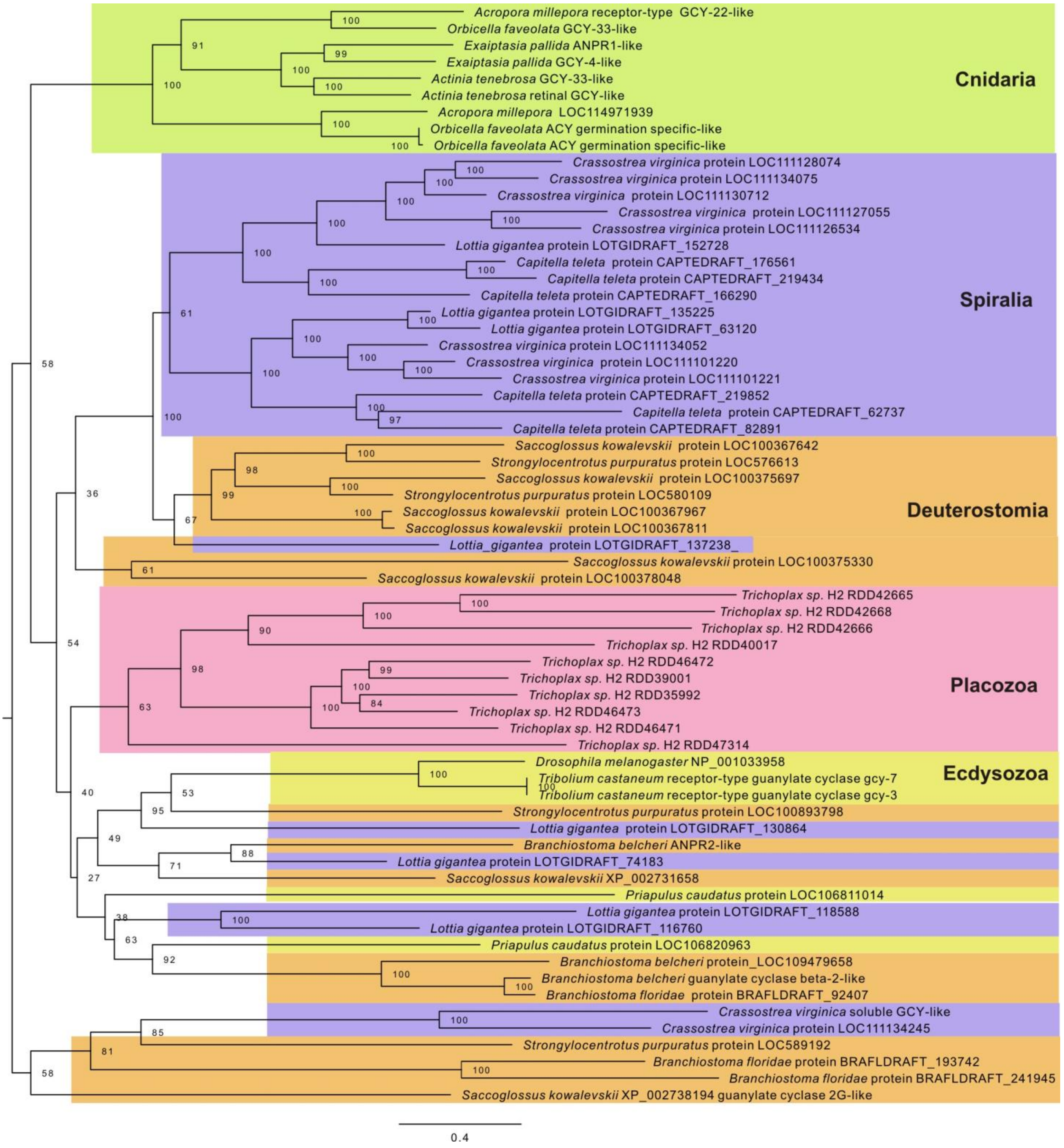

Figure 2S. Maximum likelihood phylogenetic tree of NIT domains containing guanylyl cyclases in placozoans (*Trichoplax* sp. H2 only), cnidarians and bilaterians. Guanylate cyclases with NIT domains are found in most animal phyla except sponges, ctenophores, vertebrates and urochordates. Extensive lineage-specific duplications are evident in placozoans, molluscs and hemichordates. 66 protein sequences were trimmed down to NIT+cyclase domains and produced an alignment 689 aa long. Alignment was analyzed in IQTREE [92] using LG+F+R6 evolution model chosen automatically with Bayesian information criterion. Tree robustness was tested with 2000 replicates of ultrafast bootstrap.
